# New Indazole Derivatives as Potential Scaffolds for the Development of Anticancer, Antiviral, and Anti-tuberculosis Chemotherapeutic Compounds

**DOI:** 10.2174/0109298673389070250822065247

**Published:** 2025-09-05

**Authors:** Anastasia Khandazhinskaya, Evgenya Kondrashova, Vera Sokhraneva, Olga Novikova, Yulia Velikorodnaya, Andrey Gorshenin, Sofya Andreevskaya, Tatiana Smirnova, Maxim Moroz, Ilya Kirillov, Irina Fedyakina, Alexandr Chizhov, Sergey Kochetkov, Elena Matyugina

**Affiliations:** 1 Laboratory of Molecular Basis of Action of Physiologically Active Compounds, Engelhardt Institute of Molecular Biology, Russian Academy of Sciences, 119991, Moscow, Russia;; 2 Laboratory of Immunology, Research Institute of Hygiene, Toxicology and Occupational Pathology, Federal Medical and Biological Agency, 400048, Volgograd, Russia;; 3 Microbiology Department, Central Tuberculosis Research Institute, 107564, Moscow, Russia;; 4 Medical School, Peoples’ Friendship University of Russia Named after Patrice Lumumba, 117198, Moscow, Russia;; 5 Laboratory of Virus Ecology, Gamaleya National Research Center for Epidemiology and Microbiology, Russian Ministry of Health, 123098, Moscow, Russia;; 6 Laboratory of Mass Spectrometry, Zelinsky Institute of Organic Chemistry, Russian Academy of Sciences, 119991, Moscow, Russia

**Keywords:** Indazole derivatives, drug design, anticancer activity, antiviral compounds, *Mycobacterium tuberculosis*, inhibition

## Abstract

**Introduction:**

Chemotherapy remains essential despite advances in immunotherapy, radiotherapy, and biological therapy. However, the wide range of chemical drugs is limited by a narrow therapeutic index, low selectivity, and the development of resistance. In this regard, new high-efficiency drugs are in extremely high demand. The indazole moiety, a scaffold found in many biologically active compounds, was selected for use in new drug design.

**Methods:**

Six new indazole derivatives were synthesized *via* Suzuki-Miyaura coupling starting from bromoindazole. Their antiviral (against influenza A and SARS-CoV-2), antibacterial (against *M. tuberculosis*), and antiproliferative activities (against neuroblastoma, glioma, leukemia cell lines) were evaluated *in vitro*. Acute toxicity was assessed in mice of both sexes *via* single intragastric administration, with toxicometric parameters and pathomorphological changes studied.

**Results:**

6-(1H-pyrazol-4-yl)-1H-indazole (**8**) suppressed the reproduction of the influenza virus at non-toxic doses to the MDCK cells and showed cytotoxicity against cancer cell lines, with an IC_50_ between 4 and 14 µM. However, it exhibited significant acute toxicity in mice (LD_50_ 40 mg/kg), causing systemic organ damage.

**Discussion:**

Derivative **8** demonstrated promising antiviral and antiproliferative activities but exhibited considerable acute toxicity *in vivo*. The antiviral efficacy, although lower than oseltamivir, is meaningful and justifies further optimization and investigation. Its antibacterial activity against *M. tuberculosis* adds to its potential as a multifunctional agent.

**Conclusion:**

While derivative **8** has shown potential as an antiviral and anticancer agent, its high toxicity highlights the need for further studies to define a safe and effective therapeutic window. Overall, the indazole scaffold remains a valuable platform for the development of new therapeutic compounds.

## 1 INTRODUCTION

**Fig. (1):**
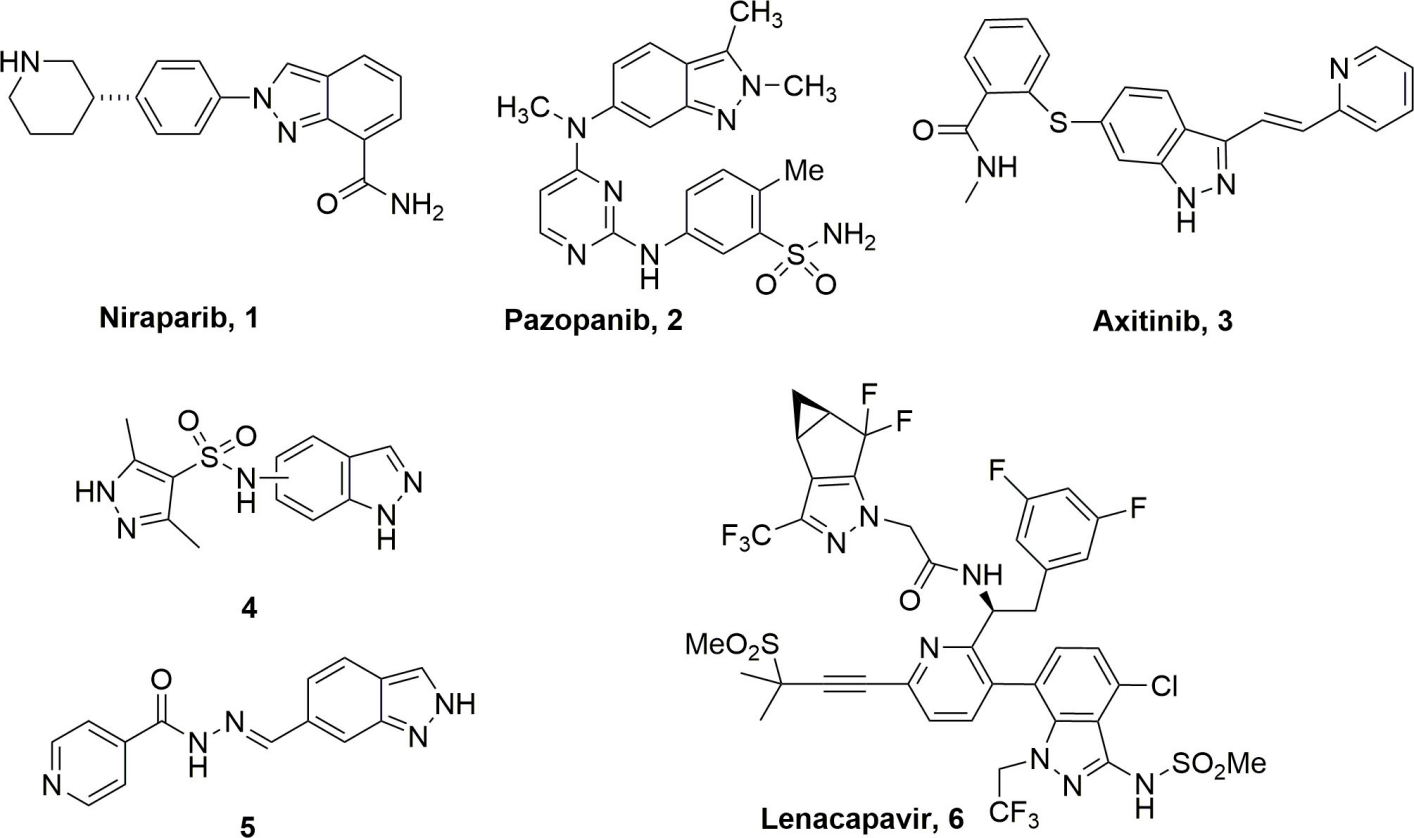
Biologically active indazole-containing drugs.

Due to their diverse biological potential, nitrogen- containing heterocycles have attracted significant scientific and practical interest for decades, which has not been vanished in recent years [1-6]. Today, N-containing heterocyclic drugs are the best-selling drugs in the world [7]. Indazole, so-called benzopyrazole or isoindazone, is a bicyclic fused ring system of pyrazole and a benzene ring. The indazole moiety is a component of many biologically active compounds, exhibiting properties such as antitumor, antifungal, anti-inflammatory, antibacterial, and anti-HIV effects [8-12]. Significant progress has been made in the development of indazole-containing drugs [8, 13-18] aimed at treating oncological diseases, which are significant medical and social problems and the leading cause of death in most countries of the world. A number of drugs containing indazole are commercially available today (Fig. **1**). Niraparib **1** is widely used to treat recurrent epithelial ovarian cancer, fallopian tube cancer or primary peritoneal cancer, breast cancer and prostate cancer [19]. Pazopanib **2** is an FDA-approved multikinase inhibitor that has been used for the treatment of renal cell carcinoma [20]. Axitinib **3** is another medication for the treatment of kidney malignancies [21]. Two anti-inflammatory drugs used in therapy, Bendazac and Benzydamine, also have analgesic action [22, 23]. Indazole sulfonamides [24], isoniazid derivatives bearing an indazole fragment [25], and 3-methyl-1-H-indazoles [26] have antibacterial properties. N-(1H-indazol-6-yl)-3,5-dimethyl-1H-pyrazole-4-sulfonamide **4** inhibits the growth of mycobacteria with a minimum inhibitory concentration (MIC) of 2 µM. An isoniazid derivative containing a 2H-indazole fragment **5** inhibited *M. tuberculosis* with an MIC of 0.11 µM (MIC for INH 0.36 µM). The indazole-containing moiety, 7-bromo-4-chloro-1H-indazol-3-amine, is part of the HIV-1 capsid inhibitor Lenacapavir **6**, developed by Gilead, which is successfully used in therapy [27]. Indazoles, bearing residues at 3- and/or 6- positions as reported, are inhibitors of fibroblast growth factor receptor kinase 1 (FGFR1), which exhibited cytotoxicity towards several cancer cell lines in nanomolar concentrations [28, 29]. The above listed drugs (Fig. **1**) are complex structures that contain indazole as a basic skeleton, substituted at 2,7- 2,5,8- 3,6- positions (anticancer drugs), with pyrazole sulfonamide residue at 5- or 6- positions (anti-tuberculosis drugs), and at 1,3,4,7- positions (anti-HIV drug). Depending on the nature and place of the substituents, the molecules exhibit different biological activities.

Despite the increasing trend of using immunotherapy, radiotherapy, and biological therapy in the treatment of diseases of various etiologies, chemotherapy remains the basis of modern medical practice. However, the wide range of chemical drugs is limited by a narrow therapeutic index, low selectivity, and the development of resistance. In this regard, the development of new chemotherapeutic drugs with high efficiency and low toxicity is still urgently needed [30].

Our study aimed to evaluate the possibility of influencing the biological activity (anticancer, antibacterial, antiviral) by varying the type and position of the substituent. Based on the published data [15, 24, 28, 29], we selected 5- and 6-positions for introducing modifications. Pyrazole, pyrimidine, pyridine, and cyclopentene with different hydrophobicity, were selected as substituents. The aim of the present research is to design and synthesize new indazole derivatives, as well as assess their antiviral (RNA-containing influenza A and SARS-CoV-2 viruses), antibacterial (*M. tuberculosis*), and antiproliferative (neuroblastoma, glioma, and leukemia) activity. Also, the acute toxicity of the lead compound was studied in laboratory mice.

## 2 MATERIALS AND METHODS

### 2.1 Chemistry

Commercially available reagents were from Aldrich, Fluka, and Acros Organics. NMR spectra were recorded on an AMX III-400 spectrometer (Bruker, Billerica, USA) with an operating frequency of 400 MHz and 300 MHz for ^1^H NMR (solvent—DMSO-d6, Me_4_Si as internal standard) and 100.6 MHz for ^13^C NMR (Figs. **S1**-**S12**). High-resolution mass spectra (HRMS) were recorded as described [31] (Figs. **S13**-**S18**).

### 2.2 General Procedure for the Synthesis of 7-12

5-Bromo-1H-indazole or 6-bromo-1H-indazole (1-1.5 mmol) was dissolved in 10 mL DME or dioxane and stirred for 20 min with 5 mol% Pd(PPh_3_)_4_ under an inert atmosphere. Then, a corresponding boronic acid or its pinacol ester, along with 10 mL of NaHCO_3>_, was added. The mixture was refluxed for 4 to 8 hours. The reaction progress was monitored by TLC in a CHCl_3_ system, and controlled by TLC in a CHCl_3_:MeOH (95:5) system. Purification was performed by column chromatography on silica gel 60 0.040-0.063 mm, eluted with a CHCl_3_: MeOH system or Hex: EtOAc system.

**5-(1H-pyrazol-4-yl)-1H-indazole (7)** was synthesized from 5-bromo-1H-indazole (200 mg, 1 mmol) and 3-(4,4,5,5-tetramethyl-1,3,2- dioxoborolan-2-yl)pyrazole (388 mg, 2 mmol) dissolved in DME. The elution system for purification was CHCl_3_:MeOH 98:2, then CHCl_3_:MeOH 95:5, then CHCl_3_:MeOH 9:1. Yield = 31.1 mg, 16.6%. ^1^H NMR (300 MHz, DMSO-d6) δ 13.00 (s, 1H), 8.04 (d, J = 6.0 Hz, 3H), 7.95 (t, J = 1.1 Hz, 1H), 7.63 (d, J = 1.6 Hz, 1H), 7.60 - 7.51 (m, 1H). ^13^C NMR (75 MHz, DMSO-d6) δ 139.2, 133.8, 125.8(2С), 125.3, 123.9, 122.2, 116.3 (2С), 110.9. HRMS, m/z: calculated for С_10_Н_8_N_4_ [М+H]^+^ 185.0822, found [М+H]^+^ 185.0827.

**6-(1H-pyrazol-4-yl)-1H-indazole (8)** was synthesized from 6-bromo-1H-indazole (300 mg, 1.5 mmol) and 3-(4,4,5,5-tetramethyl-1,3,2- dioxoborolan-2-yl)pyrazole (582 mg, 3 mmol) dissolved in DME. The elution system for purification was CHCl_3_:MeOH 98:2 and then CHCl_3_: MeOH 95:5. Yield = 110 mg, 39.3%. ^1^H NMR (300 MHz, DMSO-d6) δ 12.98 (s, 2H), 8.14 (m, 2H), 8.01 (s, 1H), 7.74 - 7.69 (m, 2H), 7.40 (dd, J = 8.4, 1.4 Hz, 1H). ^13^C NMR (75 MHz, DMSO-d6) δ 141.1, 133.9, 131.4, 122.0, 121.9, 121.3, 119.7, 105.7. HRMS, m/z: calculated for С_10_Н_8_N_4_ [М+H]^+^ 185.0822, found [М+H]^+^ 185.0830.

**5-(pyrimidin-5-yl)-1H-indazole (9)** was synthesized from 5-bromo-1H-indazole (200 mg, 1 mmol) dissolved in dioxane and pyrimidine-5-boronic acid (250 mg, 2 mmol) dissolved in methanol. The elution system for purification was CHCl_3_:MeOH 95:5. Yield = 25 mg, 12.6%. ^1^H NMR (300 MHz, CD_3_OD) δ 9.13 (s, 1H), 9.03 (s, 2H), 8.14 (m, 1H), 8.02 (dd, J = 1.7, 0.9 Hz, 1H), 7.70 (dt, J = 8.8, 1.0 Hz, 1H), 7.62 (dd, J = 8.7, 1.7 Hz, 1H). ^13^C NMR (75 MHz, CD_3_OD) δ 156.0, 154.9(2C), 140.3, 135.4, 134.3, 126.4, 125.7, 123.6, 119.7, 111.6. HRMS, m/z: calculated for С_11_Н_8_N_4_ [М+H]^+^ 197.0822, found [М+H]^+^ 197.0819.

**5-(pyridin-3-yl)-1H-indazole (10)** was synthesized from 5-bromo-1H-indazole (200 mg, 1 mmol) and 3-(4,4,5,5-tetramethyl-1,3,2-dioxoborolan-2-yl)-pyridine (615 mg, 1.5 mmol) dissolved in DME. The elution system for purification was CHCl_3_:MeOH 98:2. Yield = 29.1 mg, 14.7%. ^1^H NMR (300 MHz, CDCl_3_) δ 8.90 (dd, J = 2.4, 0.9 Hz, 1H), 8.60 (dd, J = 4.8, 1.6 Hz, 1H), 8.15 (s, 1H), 7.95 - 7.91 (m, 2H), 7.64 - 7.58 (m, 2H), 7.39 (ddd, J = 8.0, 4.9, 0.9 Hz, 1H). ^13^C NMR (75 MHz, CDCl_3_) δ 148.2, 147.8, 139.9, 137.2, 134.97, 134.7, 130.8, 126.5, 123.9, 123.7, 119.4, 110.7. HRMS, m/z: calculated for С_12_Н_9_N_3_ [М+H]^+^ 196.0869, found [М+H]^+^ 196.0870.

**6-(pyrimidin-5-yl)-1H-indazole (11)** was synthesized from 6-bromo-1H-indazole (200 mg, 1 mmol) and 3-(4,4,5,5-tetramethyl-1,3,2-dioxoborolan- 2-yl)-pyridine (615 mg, 1.5 mmol) dissolved in DME. The elution system for purification was CHCl_3_:MeOH 95:5. Yield = 35 mg, 17.6%. ^1^H NMR (300 MHz, CD_3_OD) δ 9.17 (s, 1H), 9.08 (s, 2H), 8.11 (d, J = 1.0 Hz, 1H), 7.93 (dd, J = 8.4, 0.9 Hz, 1H), 7.80 (dt, J = 1.8, 1.0 Hz, 1H), 7.41 (dd, J = 8.4, 1.5 Hz, 1H). ^13^C NMR (75 MHz, CD_3_OD) δ 156.5, 155.2(2C), 140.7, 135.3, 133.7, 132.3, 123.2, 122.1, 119.9, 108.9. HRMS, m/z: calculated for С_11_Н_8_N_4_ [М+H]^+^ 197.0822, found [М+H]^+^ 197.0844.

**6-(cyclopenten-1-en-1-yl)-1H-indazole (12)** was synthesized from 6-bromo-1H-indazole (300 mg 1.5 mmol) and 2-(4,4,5,5-tetramethyl-1,3,2-dioxoborolan- 2-yl)-cyclopenten (1.5 equiv, 443 mg, 2.5 mmol) dissolved in DME. The elution system for purification was Hex:EtOAc 3:1. Yield = 97 mg, 35%. ^1^H NMR (300 MHz, DMSO-d6) δ 12.98 (s, 1H), 8.01 (s, 1H), 7.68 (d, J = 8.4 Hz, 1H), 7.47 - 7.29 (m, 2H), 6.37 (p, J = 2.2 Hz, 1H), 2.74 (td, J = 7.5, 2.2 Hz, 2H), 2.58 - 2.49 (m, 4H), 1.99 (q, J = 7.5 Hz, 2H). ^13^C NMR (75 MHz, DMSO-d6) δ 142.7, 140.9, 134.6, 133.7, 127.2, 122.2, 120.6, 119.4, 106.9, 33.5, 33.4, 23.2. HRMS, m/z: calculated for С_12_Н_12_N_2_ [М+H]^+^ 185.1073, found [М+H]^+^ 185.1082.

### 2.3 Virology Assays

Vero E6 cells and MDCK cells were taken from the Russian National Collection of Cell Cultures at the N.F. Gamaleya National Research Center for Epidemiology and Microbiology, of the Ministry of Health of the Russian Federation (Moscow, Russia). These cells were cultivated in Eagle MEM medium (Chumakov FSC R&D IBP RAS) supplemented with a double set of amino acids, 5% fetal calf serum (Gibco), 10 mM glutamine, and 4% gentamicin. Every 2-3 weeks, they were checked for mycoplasma contamination by standard PCR.

The test compounds were dissolved in DMSO and then diluted in the appropriate media before each experiment.

Antiviral experiments were carried out as described earlier [31], according to the protocols recommended by the World Health Organization (WHO) [32].

### 2.4 Determination of the Cytotoxic effect of Substances in the Culture of MDCK Cells

The MDCK cell culture at a concentration of 18,000 cells/0.1 mL was cultivated for 24h at 37°C in an atmosphere of 5% CO_2_. Multiple dilutions of solutions of all compounds were prepared in 24-well plates by titration into 8 wells, starting from a dilution of 1:10 to 1:1280. The resulting serial dilutions were transferred to the wells of the test plates with cells. Each point was tested in 4 parallel wells. The working medium was added to the wells of the cell control. After incubating the cells with the preparations for 66 hours at 37°C in a 5% CO_2_ atmosphere, the state of the cell monolayer was visually assessed. Then, the culture medium was removed from the plates, and 100 μL of the medium and 20 μL of the MTS solution were added to the cell culture monolayer in each well. After incubation for 3 hours at 37°C, the results were recorded on a BIO-RAD automatic reader at a wavelength of 490 nm. Reference filter - 630 nm.

### 2.5 Study of the Effect of Compounds on the Infectious Titer of the Influenza Virus in MDCK Cell Culture

#### 2.5.1 Influenza Virus Assay

Tests on MDCK cells were performed as described [33].

#### 2.5.2 SARS-CoV-2 Assays

Vero E6 cells were seeded on 96-well plates at a density of 1.2 × 10^4^ cells/well, infected with serial dilutions of SARS-CoV-2, and incubated with compounds as described earlier [33]. The virus titer was assessed by the determination of the endpoint dilution at which it exhibits 50% of its maximal cytopathic effect (TCID_50_). The antiviral activity was expressed as a logarithmic change (∆lg_max_) in the TCID_50_ value in the presence of a compound compared with the DMSO-treated control. Each compound was tested in quadruplicate.

### 2.6 Antituberculosis Tests

The antituberculosis activity of the compounds **7-12** was determined by the proportion method based on the growth dynamics of the laboratory virulent strain *M. tuberculosis* H37Rv culture on the liquid medium Middlebrook 7H9 in the automatic system for detection of mycobacterial growth and drug susceptibility testing of mycobacteria BACTEC MGIT 960 (BD, USA) as described earlier [34]. Before conducting the studies, the *M. tuberculosis* H37Rv culture was standardized for the number of CFU and growth phase as described previously [35]. Tested compounds were dissolved in DMSO/H_2_O (1:1, *v/v*). The standardized suspension of mycobacteria (5x10^6^ CFU/mL) was introduced in a volume of 500 μl into MGIT tubes and cultured for 42 days in the presence of the studied compounds at concentrations of 10, 20, 40 and 60 μg/mL, without drugs (negative control) and in the presence of rifampicin and isoniazid at critical concentrations (positive control). In addition, a series of dilutions of the standardized suspension of mycobacteria were cultured under the same conditions in the ratio (CFU: medium) of 1:99, 1:9, 1:3, and 1:1. Each concentration of the compounds, as well as control samples, was studied in triplicate. The level of bacteriostatic activity of the compounds was determined by comparing the parameters of culture growth recorded by the BACTEC MGIT 960 software in samples containing the test compounds and in samples with dilutions of the mycobacterial suspension: if on the day when growth at the level of 400 growth units (GU) was recorded in tubes with a culture diluted in a ratio of 1:99, 1:9, 1:3 and 1:1, and less than 100 GU was recorded in the test samples, then this concentration of the compound inhibited culture growth by at least 99%, 90%, 75% and 50%, respectively. The minimum concentrations at which the corresponding growth inhibition was achieved were designated as MIC(99), MIC(90), MIC(75), and MIC(50) [36].

### 2.7 Statistical Data Processing

To obtain statistically reliable results, the experiments were performed 2-3 times. The values of CC_50_ and IC_50_ were calculated using generally accepted methods for biological research, as implemented in the Microsoft Excel 5.0 and GraphPad Prism 6.01 software packages. The working model for CC_50_ analysis was a 4-parameter logistic curve equation (menu items “Nonlinear regression” - “Sigmoidal dose-response (variable slope)”). For IC_50_ analysis, a 4-parameter logistic curve equation was adopted (menu items “Nonlinear regression” - “log (inhibitor) *vs.* response (variable slope)”). Based on the obtained data, the selectivity index (SI) was calculated using the equation: SI = CC_50_/ IC_50_.

### 2.8 General Procedure for Cytotoxicity Testing on Cell Cultures

Leukemia cells from ATCC K562 (CCL-243) and HL60 (CCL-240) were cultured using RPMI 1640 medium (Servicebio) supplemented with 10% fetal bovine serum (FBS) and 2 mM L-glutamine. Cell cultures were incubated at 37°C in a 5% CO_2_ atmosphere. Neuroblastoma cells SH-SY5Y (CRL-2266) from ATCC were cultured in DMEM medium (Servicebio) containing 10% fetal calf serum (FBS) at 37°C in an atmosphere of 5% CO_2_. The low-passage primary glioblastoma multiforme cell culture GBM5522 was previously described [37]. Cells were cultured in DMEM (Servicebio) containing 10% fetal bovine serum (FBS) at 37°C in an atmosphere of 5% CO_2_. The cells were plated in 96-well plates at a density of 1x10^4^ cells/well. The test substances were then added in concentrations ranging from 0.8 to 100 μM. DMSO at a concentration of 1% was used as a control, corresponding to the percentage of DMSO when the highest concentration of the drug was added. The total well volume was 150 μl. The cells were incubated for 48 hours at 37°C in a 5% CO_2_ atmosphere. Resazurin was added in a concentration of 10 μl of a 0.5 mg/mL solution in phosphate-buffered saline. The incubation was carried out under the same conditions for 4 hours, and then the fluorescence level was measured at an excitation wavelength of 560 nm and an emission wavelength of 590 nm using a CLARIOstar multimodal microplate reader (BMG Labtech, Germany). The data obtained for each concentration were normalized to the control, and the IC_50_ was calculated using the nonlinear regression method. The minimum number of repetitions for each concentration was three. The GraphPad Prism software v.9.5.1 (GraphPad Software, San Diego, USA) was used to calculate the IC_50_ and plot the percentage of living cells versus the concentration of the drug.

### 2.9 Toxicity of Compound 8 for White Outbred Mice

The toxicity of the compound was studied according to published procedure [38] in compliance with the guidelines [39].

## 3 RESULTS AND DISCUSSION

**Scheme 1:**
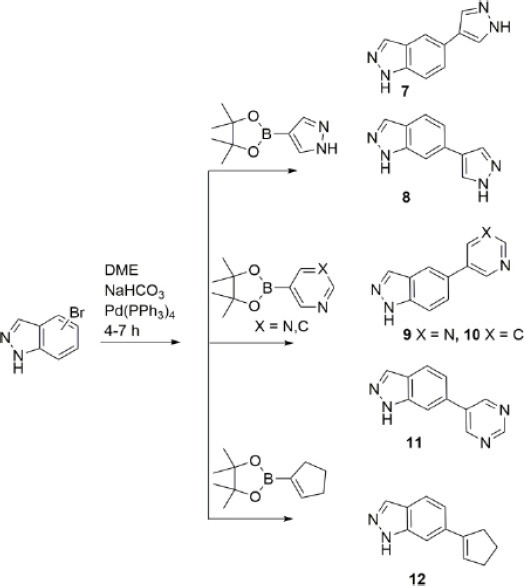
Synthesis of 5(6)-substituted indazole derivatives **7-12**.

Indole and indazole derivatives bearing phenyl, pyridine, thiophene or furan residues in the 6-position have been described previously [28]. The compounds exhibited significant antitumor properties due to the inhibition of fibroblast growth factor receptor kinase 1 (FGFR1). In this work, new 5(6)-substituted indazole derivatives **7-12** were obtained in order to study their biological properties. The C-C bond formation between indazole and the cyclic/heterocyclic residues occurs *via* a cross-coupling reaction (Scheme **1**) [40, 41].

Six new compounds bearing a cyclic/heterocyclic residue were obtained starting from 5- or 6-bromoindazole (Scheme **1**) using the Suzuki-Miyaura reaction with palladium catalyst [40-42]. The compounds were isolated by column chromatography on silica gel using a Chloroform: Methanol (95:5) or Hexane: Ethyl acetate (4:1) system, and, if necessary, were repurified using preparative layer chromatography (PLC). The yield of the products was 12-40%.

The biological properties of the new compounds were studied against influenza virus and coronavirus, as well as on a panel of tumor cells, and for their ability to inhibit the growth of *Mycobacterium tuberculosis*.

The effect of non-toxic concentrations of compounds **7**-**12** on the reproduction of the influenza virus in MDCK cells was studied using the human influenza virus A/California/7/2009 (H1N1)pdm09. Each of the drug concentrations was tested in 2 parallel wells. The antiviral activity of the tested derivatives was determined by the decrease in the infectious titer of the influenza virus in MDCK cell culture, as measured by the cytopathic effect (CPE), and in the hemagglutination reaction (HAR). Oseltamivir phosphate ((3R,4R,5S)-4-acetamido-5-amino-3-(1-ethylpropo- xy)-1-cyclohexene-1-carboxylic acid ethyl ester phosphate salt from F. Hoffmann-La Roche Ltd, Switzerland) was used as a positive control. The compound was dissolved in sterile distilled water for the *in vitro* experiments.

The antiviral activity of the synthesized compounds against SARS-CoV-2 was also studied. Changes in viral RNA during viral replication in the medium (to measure the decrease in virion production) in the presence of each compound taken at a fixed concentration (∆log_10_(RNA)), and the inhibitory concentration IC_50_ were calculated. Table **1** presents summary data on the antiviral activity of the studied compounds **7-12**.

None of the tested compounds has shown significant activity against SARS-CoV-2. Compound **10** slightly inhibited the reproduction of influenza A/California/7/2009 (H1N1)pdm09 virus in the MDCK cell culture, reducing the infectious titer by a maximum of 1.5 logTCID_50_. Compound **8** inhibited the replication of influenza A/California/7/2009 (H1N1)pdm09 virus in the MDCK cell culture, reducing the infectious titer by a maximum of 2.0 logTCID_50_ with the therapeutic and prophylactic regimen of compound administration 1 hour before the infection. Chemotherapeutic index SI was 55. Compound **8** was significantly inferior to the control oseltamivir in terms of activity and selectivity index; however in accordance with modern methods of searching for drugs for influenza [39], the demonstrated activity was significant and allowed us to consider compound **8** as a candidate for further study. Moreover, compound **8** and the control oseltamivir belong to different classes of chemical compounds and most likely act on different targets, which complicates their direct comparison.

**Fig. (2):**
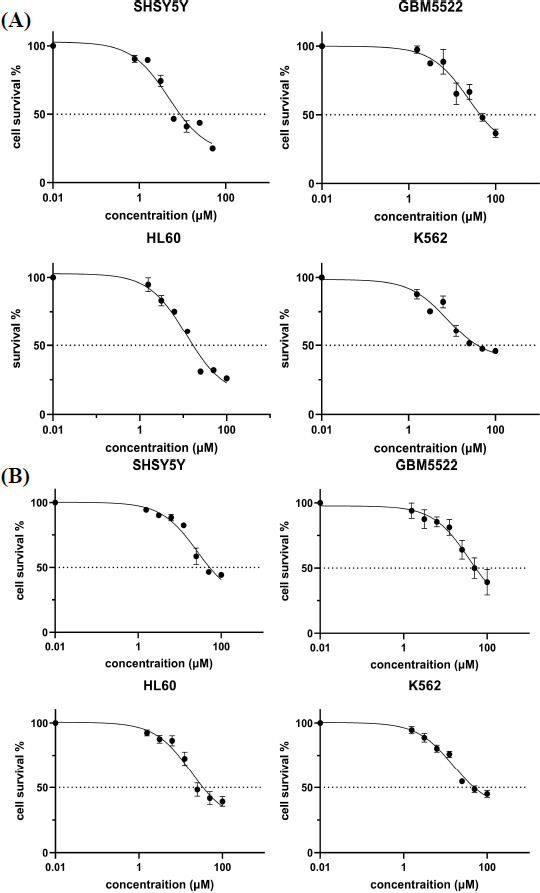
Survival of cancer cells after treatment with compounds **7 (A)** and **8 (B)**. Examples of curves are given, reflecting the percentage of living cells SH-SY5Y, GBM5522, HL60, and K562 depending on the concentration of compounds **7 (A)** and **8 (B)**. The curves were obtained by the method of nonlinear regression.

Cytotoxicity screening of the synthesized indazole derivatives was performed on the cell line of neuroblastoma SH-SY5Y, lymphoblastic cells K-562, promyeloblastic cells HL-60, low-passage variant of highly differentiated multiform glioblastoma GBM5522 (Table **2**). As a result of cytotoxicity assessment on the above-mentioned cell lines, compound **8** showed the best activity (IC_50_ 4-14 µM) (Fig. **2**), compounds **7** and **10** showed moderate activity. Compounds **9**,**11** and **12** were inactive (Table **2**).

The antimycobacterial action of compounds **7-12** was studied by the dynamics of the growth of the laboratory strain *M.tuberculosis* H37Rv culture in the enriched liquid Middlebrook 7H9 medium.

The results of the determination of antituberculosis activity of indazole derivatives are presented in Table **3**. Compounds **7** and **8** inhibited the growth of 50% of the *M.tuberculosis* population *in vitro* at a concentration of 40 μg/mL (217 µM) and of 75% the *M.tuberculosis* population at a concentration of 60 μg/mL (326 µM). Compounds **9** and **12** were more active against the *M*. *tuberculosis* test-strain and, at a concentration of 40 μg/mL (203.9 and 217.1 μM*),* inhibiting the growth of 75% and 90% of the population, respectively. The least active was compound **11**, which inhibited the growth of 50% of the mycobacterial population at a concentration of 60 μg/mL (306 µM). Compound **10** did not affect the growth of the *M.tuberculosis* H37Rv culture: the growth parameters of the culture in the presence of the specified compound, even at the maximum tested concentration, did not differ from the control without the drug.

The growth of *M. tuberculosis* H37Rv culture in the presence of critical concentrations of control drugs, rifampicin and isoniazid, was not observed, which indicated the validity of the obtained results. The MIC of the tested compounds against *M. tuberculosis* was significantly higher than the critical concentrations of the control drugs (0.5 μg/mL (0.61 µM) for rifampicin and 1 μg/mL (0.73 µM) for isoniazid). However, in the case of low toxicity of the compounds and, accordingly, a high therapeutic index, such a high MIC may not be an obstacle to further consideration of the compounds as potential agents for anti-tuberculosis therapy. An example is pyrazinamide, the critical concentration of which is 100 μg/mL (812 µM), and it is widely used in the treatment of tuberculosis [43].

The obtained data indicate that the new indazole derivatives are promising biologically active substances and warrant further development. In this regard, one representative of the group, lead compound **8**, was studied for acute toxicity in non-rodent mice. White outbred mice of both sexes, weighing 25-30 g and aged 12-13 weeks, were used as biomodels. The animals were obtained from the nursery of the Federal State Unitary Enterprise «Research Institute for Hygiene, Toxicology and Occupational Pathology» of the Federal Medical and Biological Agency of Russia. The total number of animals was 20 heads. The experimental and control groups consisted of 4 animals with an equal number of males and females.

The toxic effect of the specified substance was studied using the described procedure [38, 39]. The substance was administered intragastrically using an atraumatic metal probe at a rate of 0.05 mL per 10 g of body weight in a dose range from 20 mg/kg to 80 mg/kg. Control animals received the solvent (99% DMSO) in the same volume in a similar way.

The initial solution of the substance was obtained ex tempore by dissolving the calculated weights in DMSO. Working solutions of lower concentrations were prepared from the initial solution by the method of successive dilutions.

After the substance was administered, the animals were continuously observed for 4 hours, then periodically for 14 days, recording clinical manifestations of intoxication and the fact of death, after which lethal doses were calculated.

Dead animals were autopsied as soon as possible after death, macroscopic manifestations of the effect of the test compound were noted, and internal organs were collected [44] for subsequent histological examination as described earlier [38, 45].

Lethal doses were calculated using the probit analysis method, as described by D.J. Finney, in Microsoft Excel 2013 on the 14^th^ day of the experiment [46].

It was found that the clinical picture of intoxication in experimental animals upon the administration of the substance at the level of lethal doses developed during the first day after administration, was characterized by convulsions, dysfunction of the hind limbs, increasing hypodynamia, and after which death occurred. Table **4** presents the results of calculating the lethal doses of the studied compound. Derivative **8** showed a high degree of toxicity in mice; LD_50_ was 40.0 mg/kg.

During necropsy of animals that died during the experiment, the following macro- and microscopic modifications were noted.

In animals that received the substance at doses of 80 mg/kg and 50 mg/kg, uncoagulated blood was found in the heart, pulmonary hyperemia was present, and blood was observed in the chest. The mucous membrane of the peritoneum was hyperemic, with blood oozing and cyanosis. The stomach was swollen, filled with yellow mucous contents, and the walls were overstretched. The liver was unevenly colored, pale, with areas of hemorrhage in a large lobe. The gallbladder was not visually determined. The wall of the small intestine was flabby, thinned, and hyperemic, with brown contents in the lumen.

**Fig. (3):**
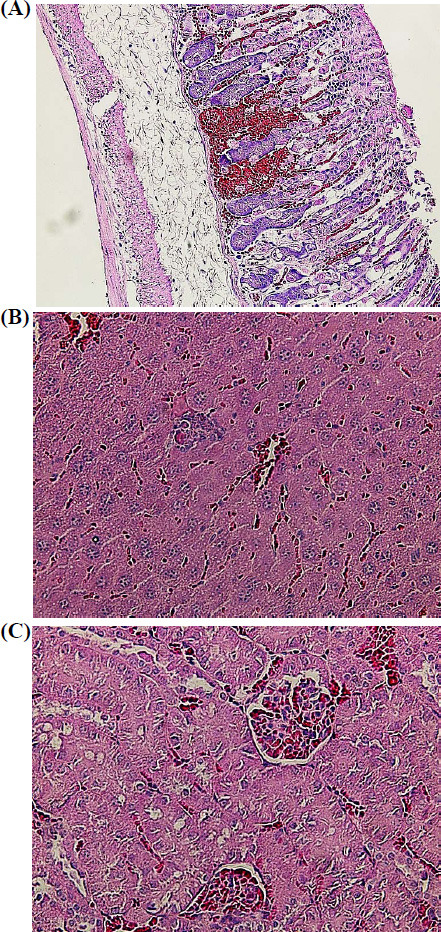
Fragments of the stomach wall (**A**), liver (**B**), and kidney (**C**) of an experimental male mouse after the administration of compound **8** at a dose of 80 mg/kg. Hematoxylin-eosin staining. Magnification 200 (**A**); 400 (**B** and **C**).

**Fig. (4):**
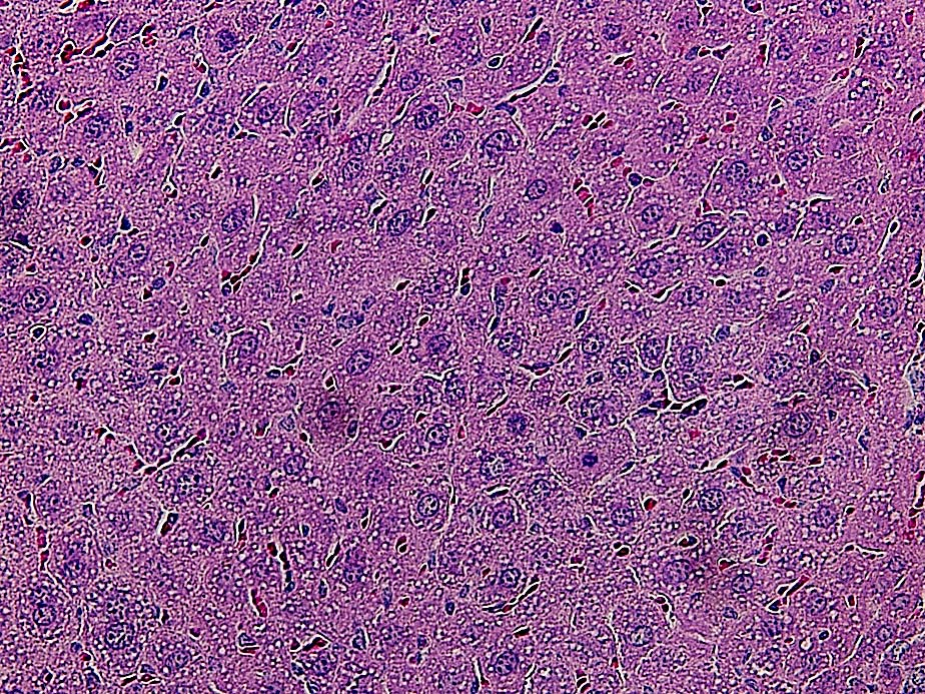
Liver fragments of an experimental female mouse after the administration of compound **8** at a dose of 80 mg/kg. Hematoxylin and eosin staining. Magnification 400.

Pathomorphological examination of organ and tissue samples from the experimental male mouse after exposure to the substance at a dose of 80 mg/kg showed focal hemorrhages in the gastric mucosa and pronounced swelling and edema of the submucosal layer (Fig. **3A**). In the liver tissue, there were histological signs of venous congestion, which were expressed by erythrocyte sludge and stasis not only in large blood vessels, but also in intralobular capillaries. Numerous vacuoles were present in the cytoplasm of hepatocytes; individual cells underwent necrotic changes (Fig. **3B**). Venous congestion was also noted in the kidneys, which was characterized by erythrocyte stasis in the capillary system of the glomeruli and peritubular space. Epithelial cells of the convoluted tubules demonstrated signs of hypertrophy, as a result of which the lumen of a significant proportion of the tubules narrowed significantly (Fig. **3C**). After the introduction of the substance at a dose of 80 mg/kg, the main pathomorphological changes in the organs of the female mouse were noted in the liver, where venous congestion, pronounced vacuolization of hepatocytes, and the presence of pyknotic nuclei in the cells were observed (Fig. **4**). Focal hemorrhages were present in the gastric mucosa, and venous congestion and hypertrophy of the epithelial cells in the convoluted tubules of the kidneys were observed.

## CONCLUSION

This study has shown that the introduction of a pyrazole at the 5-position (compound **8**) or pyridine at the 6-position (compound **10**) to the indazole skeleton results in the manifestation of activity against influenza A. Indazole derivatives synthesized with other substituents at position **5** or **6**, did not exhibit antiviral activity. Compound **8** demonstrated reliable suppression of influenza virus A/California/7/2009 (H1N1)pdm09 *in vitro*, using a therapeutic and prophylactic scheme that involved adding the compound in non-toxic doses to the MDCK cell culture. The antiviral activity of compound **8** is concentration-dependent. Indazole derivatives with pyrazole or pyridine fragments in the 5- or 6-position, namely 5-pyrazolylindazole **7**, 6-pyrazolylindazole **8**, and 5-pyrimidylindazole **10**, showed significant cytotoxicity towards cancer cell lines. Compound **8** showed maximum cytotoxicity against SH-SY5Y neuroblastoma cells, K-562 lymphoblastic cells, HL-60 promyeloblast cells, and GBM5522 low-passage highly differentiated multiform glioblastoma cells. The IC_50_ value ranged from 4 to 14 µM. There are drugs actively used in the therapy of malignant hematologic diseases with comparable cytotoxicity. For example, etoposide: the range of half-maximal effective concentrations of this drug added to continuous leukemia cell lines is about 10-100 µM [47, 48]. Among the compounds studied, the best antibacterial activity was found for 6-cyclopentenyl indazole **12** and 5-pyrimidinyl indazole **9**, which neither showed antiviral nor anticancer activity. And 5-pyrimidyl indazole **10**, which has antiviral activity and cytotoxicity against cancer cells, on the contrary, was observed to be completely devoid of the ability to suppress the growth of mycobacteria. The obtained data indicate that the indazole scaffold is of great interest for further development, as varying the location and structure of the heteroaryl substituent allows to control the biological activity of its derivatives. In this regard, one representative of the group, lead compound **8**, was studied for acute toxicity in non-rodent mice of both sexes. Derivative **8** showed a high degree of toxicity in mice, with an LD_50_ of 40.0 mg/kg. To compare, nonlethal doses of etoposide used in mouse model studies are ~ 50 mg/kg [49, 50]. Thus, additional efforts are required to improve selectivity and therapeutic index.

## Figures and Tables

**Table 1:** Results of the action of compounds 7-12 on the reproduction of influenza viruses A/California/07/2009 pdm and SARS-CoV-2.

**Comp.**	**Influenza Viruses A/California/07/2009 pdm, MDCK**				**SARS-CoV-2, Vero E6**	
	**Δlg_max_^1^**	**IC_50_^2^, µM**	**CC_50_^3^, µM**	**SI^4^**	**Δlg_max_**	**IC_50_, µM**
7	0	>20	100	<5	0.25	>50
8	2.0	6	330	55	0.75	>6
9	1.0	>100	1050	<10	0	>50
10	1.5	25	610	24,4	0.75	>25
11	0.5	>200	1120	<5	0	>200
12	0.25	>2	4	<2	0.5	>3
Oseltamivir	6.0	0.61	820	500	-	-

**Note:** ^1^ logarithmic change in the TCID_50_ (Tissue Culture Infectious Dose) value in the presence of a compound compared with DMSO-treated control; ^2^ IC_50_, concentration of a compound at which it reduced TCID_50_ by 50%; ^3^ CD_50_ (cytotoxic dose), concentration of a compound at which it reduced the cell number by 50%; ^4^ SI (selectivity index), the ratio of CD_50_ to IC_50_.

**Table 2:** Cytotoxicity of compounds 7-12 on the tumor cell cultures.

Comp.	SH-SY5Y	GBM5522	K562	HL60
	**IC_50_^1^, µM**			
7	15.5±5.3	36.8±2.0	24.4±3.1	23.7±5.0
8	4.2±0.9	13.8±1.1	6.7±0.8	9.9±0.8
9	>100	>100	>100	>100
10	14.3±5.0	95.4±5.2	5.5±1.5	>100
11	>100	>100	>100	>100
12	>100	>100	22.3±4.3	>100

**Note:** ^1^ The half-maximal inhibitory concentrations.

**Table 3:** Bacteriostatic activity of indazole derivatives against a laboratory virulent strain *M.tuberculosis* H37Rv.

**Comp.**	**Concentrations studied, μg/mL (µМ)**	**MIC^1^, μg/mL (µМ)**			
		**50**	**75**	**90**	**99**
7	10, 20, 40, 60 (54, 109, 217, 326)	40 (217)	60 (326)	-	-
8	10, 20, 40, 60 (54, 109, 217, 326)	40 (217)	60 (326)	-	-
9	10, 20, 40, 60 (51, 102, 204, 306)	40 (204)	40 (204)	-	-
10	10, 20, 40 (51, 102, 205)	-	-	-	-
11	10, 20, 40, 60 (51, 102, 204, 306)	60 (306)	-	-	-
12	10, 20, 40 (54, 109, 217)	40 (217)	40 (217)	40 (217)	-

**Note:** ^1^Minimal concentration of compounds exhibiting 50, 75, 90, and 99% inhibition in mycobacterial growth.

**Table 4:** Calculation of lethal doses of compound 8 for mice after a single intragastric administration.

**Studied Dosages, mg/kg**	**Number of Animals in the Group/Number of Dead**	**Estimated Lethal Dose Values, mg/kg**			
		**LD_10_^1^**	**LD_16_^1^**	**LD_50_^1^**	**LD_84_^1^**
20 40 50 80	4/0 4/2 4/3 4/4	26,2	28,8	40,0 (33,6…47,6)	55,6

**Note:** ^1^LD_10_, LD_16_, LD_50_, and LD_84_ - doses causing death in 10, 16, 50, and 84% of experimental animals upon intragastric administration on day 14 of observation.

## Data Availability

All data generated or analyzed during this study are included in this published article.

## LIST OF ABBREVIATIONS

CPE: Cytopathic Effect
CD_50_ (Cytotoxic Dose): The concentration of a compound at which it reduces the cell number by 50%
DMSO: Dimethyl Sulfoxide
HAR: Hemagglutination Reaction
IC_50_: Concentration of a compound at which it reduced Tissue Culture Infectious Dose (TCID_50_) by 50%
LD_16_: Dose causing death of 16% of experimental animals when administered intragastrically on day 14 of observation, mg/kg
LD_50_: Dose causing death of 50% of experimental animals when administered intragastrically on day 14 of observation, mg/kg
LD_84_: Dose causing death of 84% of experimental animals when administered intragastrically on day 14 of observation, mg/kg
MIC: Minimal concentration of compounds exhibiting 50, 75, 90, and 99% inhibition in mycobacterial growth
PLC: Preparative layer chromatography
SI (selectivity index): The ratio of CD_50_ to IC_50_
