# Supplementary material for: New Indazole Derivatives as Potential Scaffolds for the Development of Anticancer, Antiviral, and Anti-tuberculosis Chemotherapeutic Compounds
Source: Curr Med Chem. 2025 Sep 5;33(5):1021–34. doi: 10.2174/0109298673389070250822065247 (PMC13223490; doi:10.2174/0109298673389070250822065247)
Supplement: Supplementary file 1 [file CMC-33-5-1021_SD1.pdf]

Supplementary Material

New Indazole Derivatives as Potential Scaffolds for the Development of Anticancer, Antiviral, and Anti-tuberculosis Chemotherapeutic Compounds

Khandazhinskaya Anastasia[1], Kondrashova Evgenya[1], Sokhraneva Vera[1], Novikova Olga[2], Velikorodnaya Yulia[2], Gorshenin Andrey[2], Andreevskaya Sofia[3], Smirnova Tatyana[3], Moroz Maxim[4], Kirillov Ilya[5], Fedyakina Irina[5], Chizhov Alexandr[6], Kochetkov Sergey[1] and Matyugina Elena[1,*]

[1]Laboratory of Molecular Basis of Action of physiologically active compounds, Engelhardt Institute of Molecular Biology, Russian Academy of Sciences, 119991, Moscow, Russia; [2]Laboratory of Immunology, Research Institute of Hygiene, Toxicology and Occupational Pathology, Federal Medical and Biological Agency, 400048, Volgograd, Russia; [3]Microbiology Department, Central Tuberculosis Research Institute, 107564, Moscow, Russia; [4]Medical school, Peoples' Friendship University of Russia Named after Patrice Lumumba, 117198, Moscow, Russia; [5]Laboratory of virus ecology, Gamaleya National Research Center for Epidemiology and Microbiology, Russian Ministry of Health, 123098, Moscow, Russia; [6]Laboratory of mass spectrometry, Zelinsky Institute of Organic Chemistry, Russian Academy of Sciences, 119991, Moscow, Russia

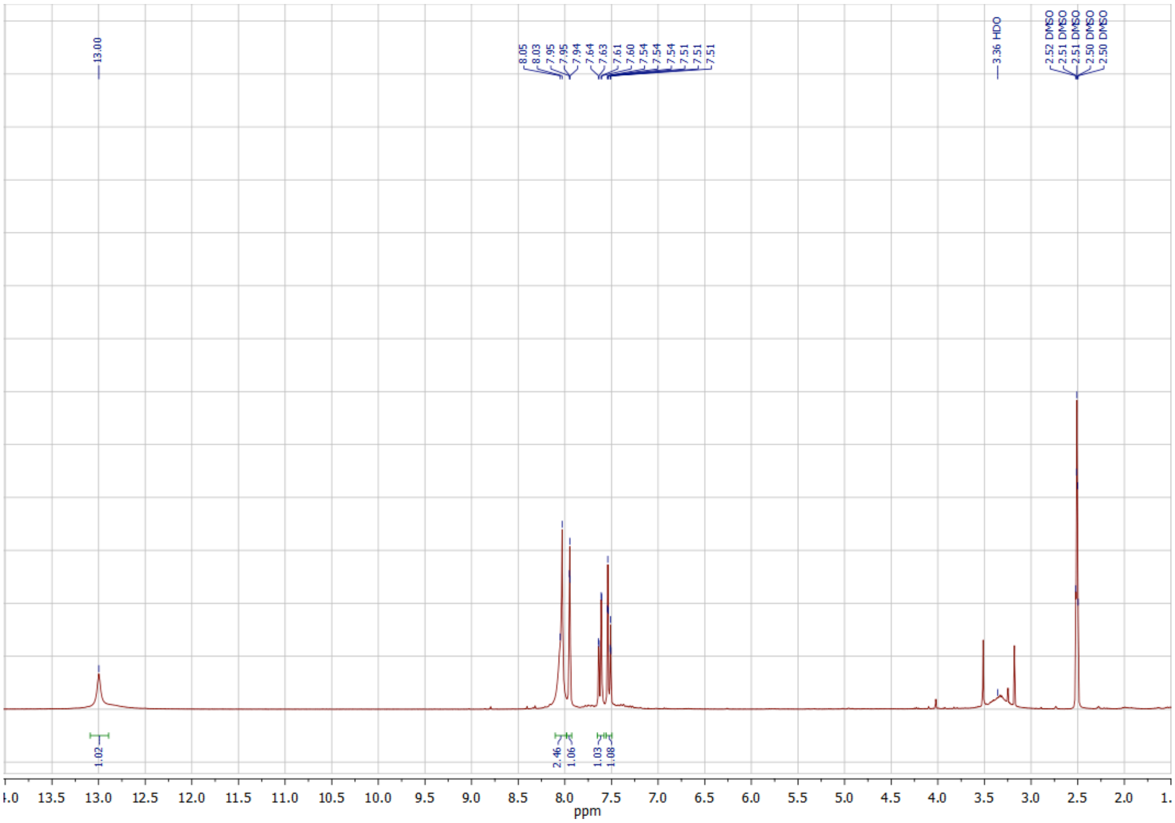

Fig. (S1). ^{1}H NMR spectrum of **7** in DMSO-d_6.

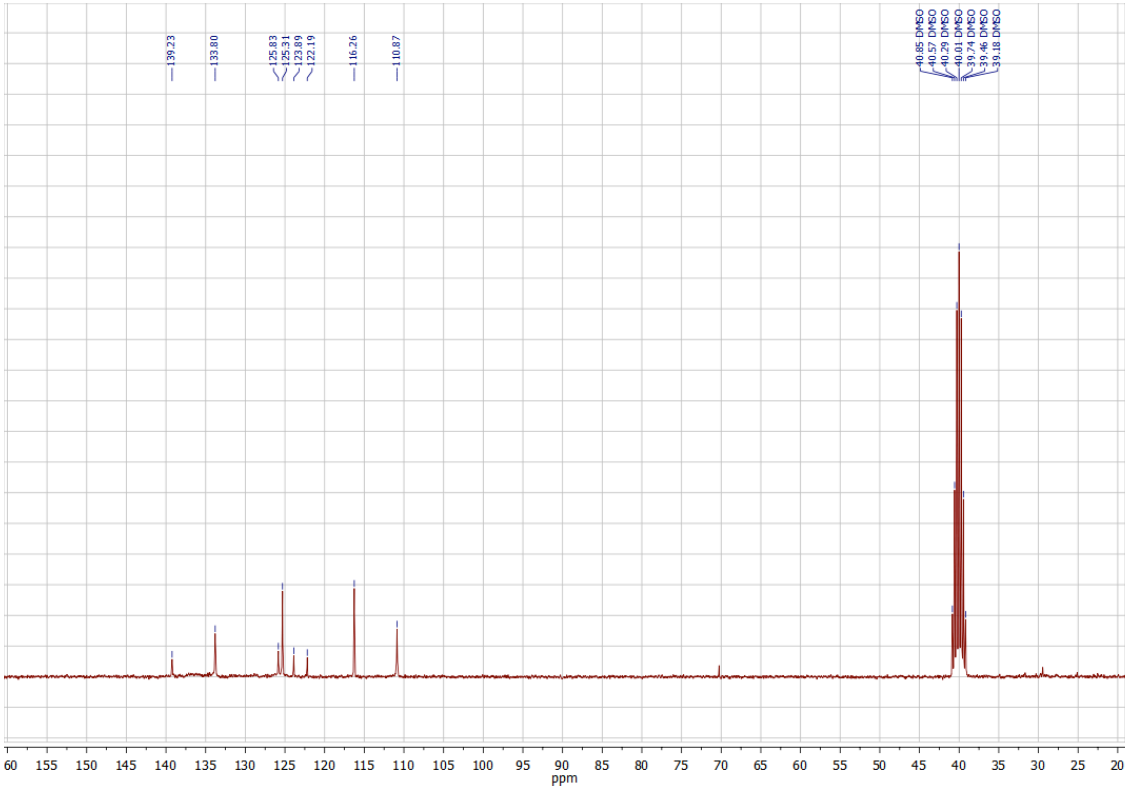

Fig. (S2). ^{13}C NMR spectrum of **7** in DMSO-d_6.

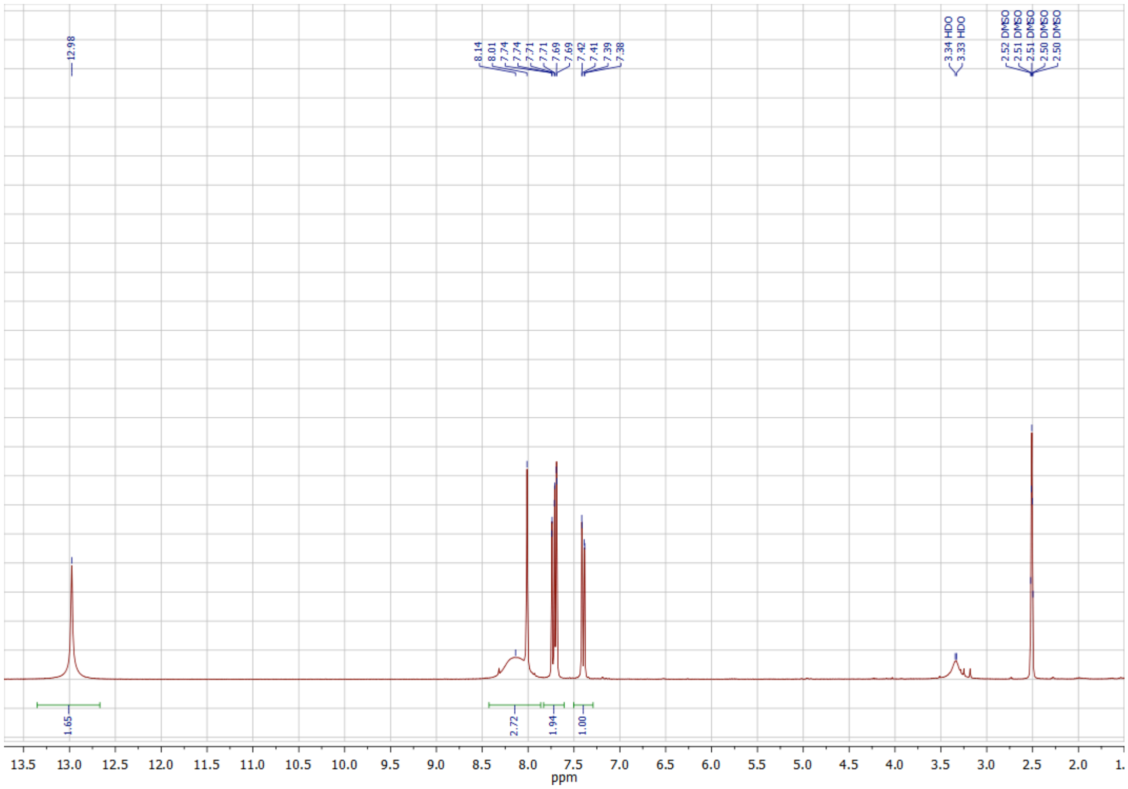

Fig. (S3). ^{1}H NMR spectrum of **8** in DMSO-d_6.

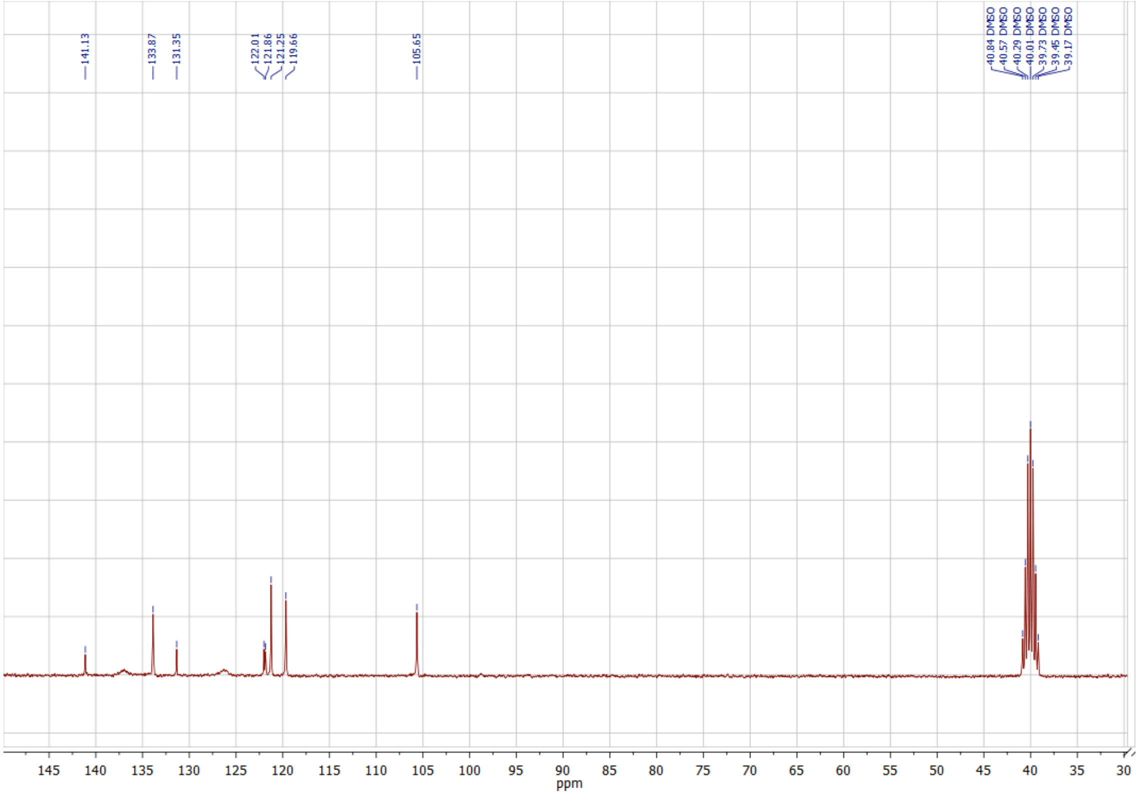

Fig. (S4). ^{13}C NMR spectrum of **8** in DMSO-d_6

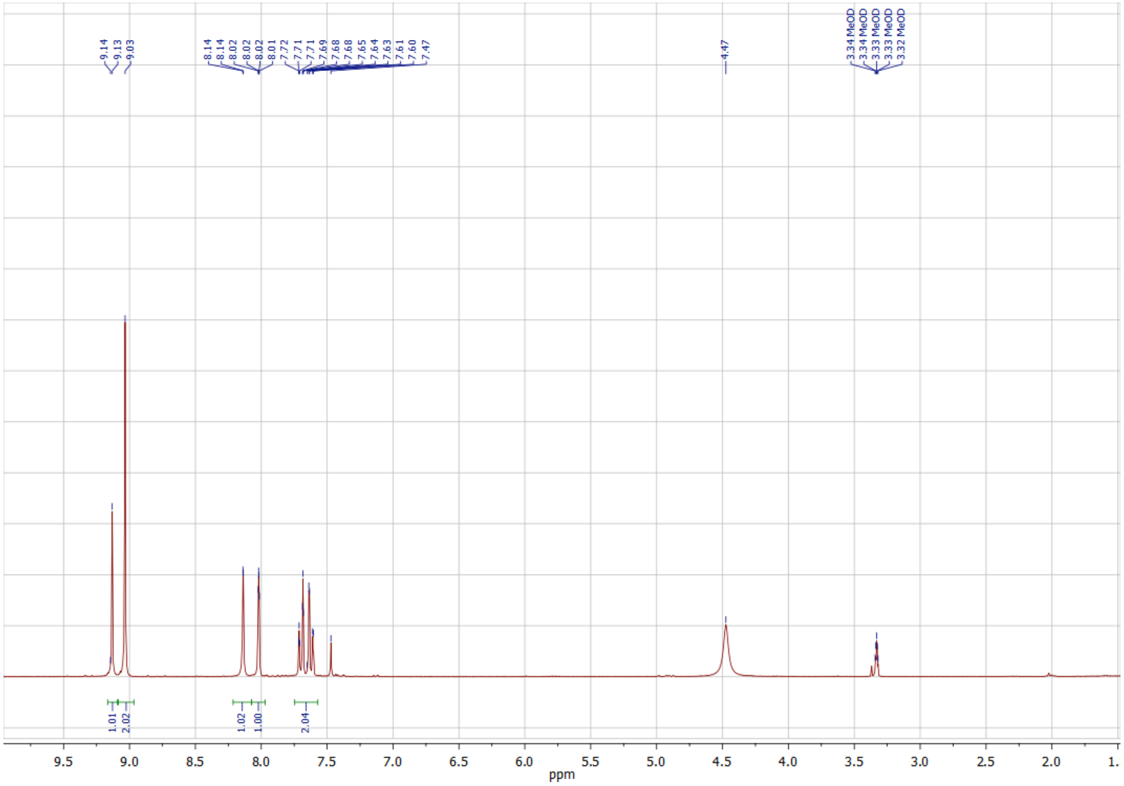

Fig. (S5). ^{1}H NMR spectrum of **9** in CH_3OD:$CDCl_3$ (1:4).

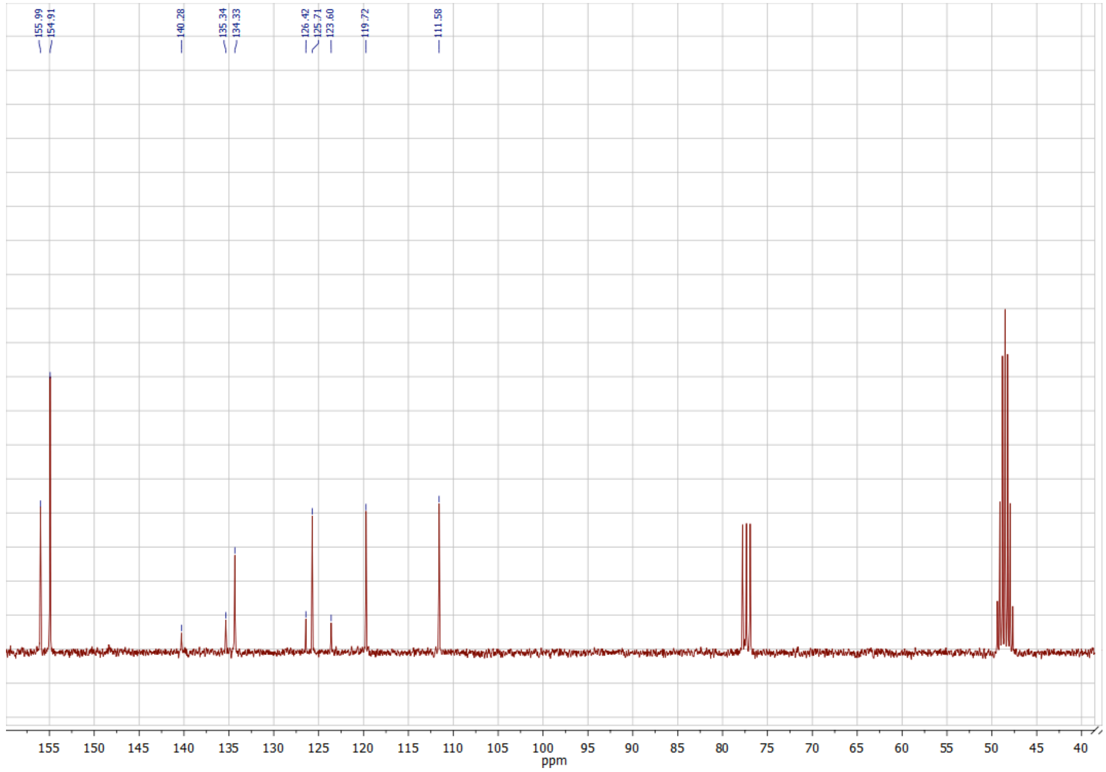

Fig. (S6). ^{13}C NMR spectrum of **9** in CH_3OD:$CDCl_3$ (1:4)

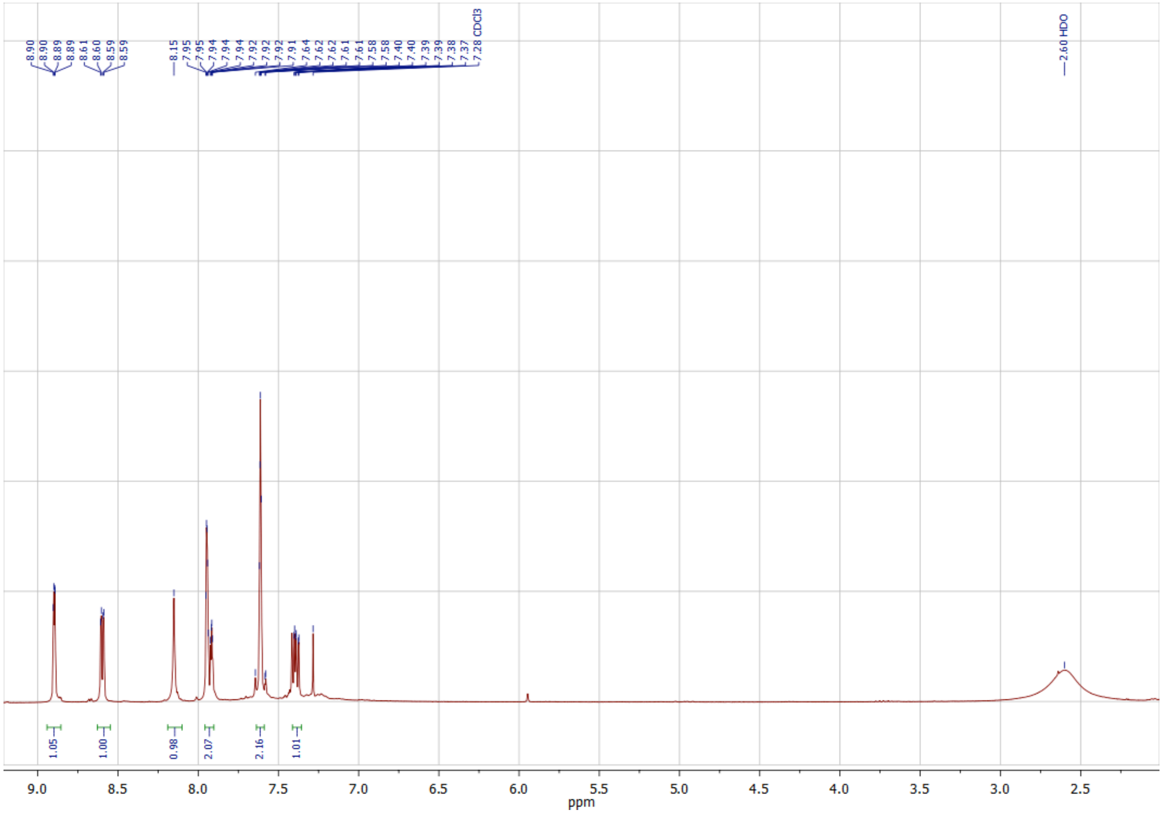

Fig. (S7). ^{1}H NMR spectrum of **10** in $CDCl_3$

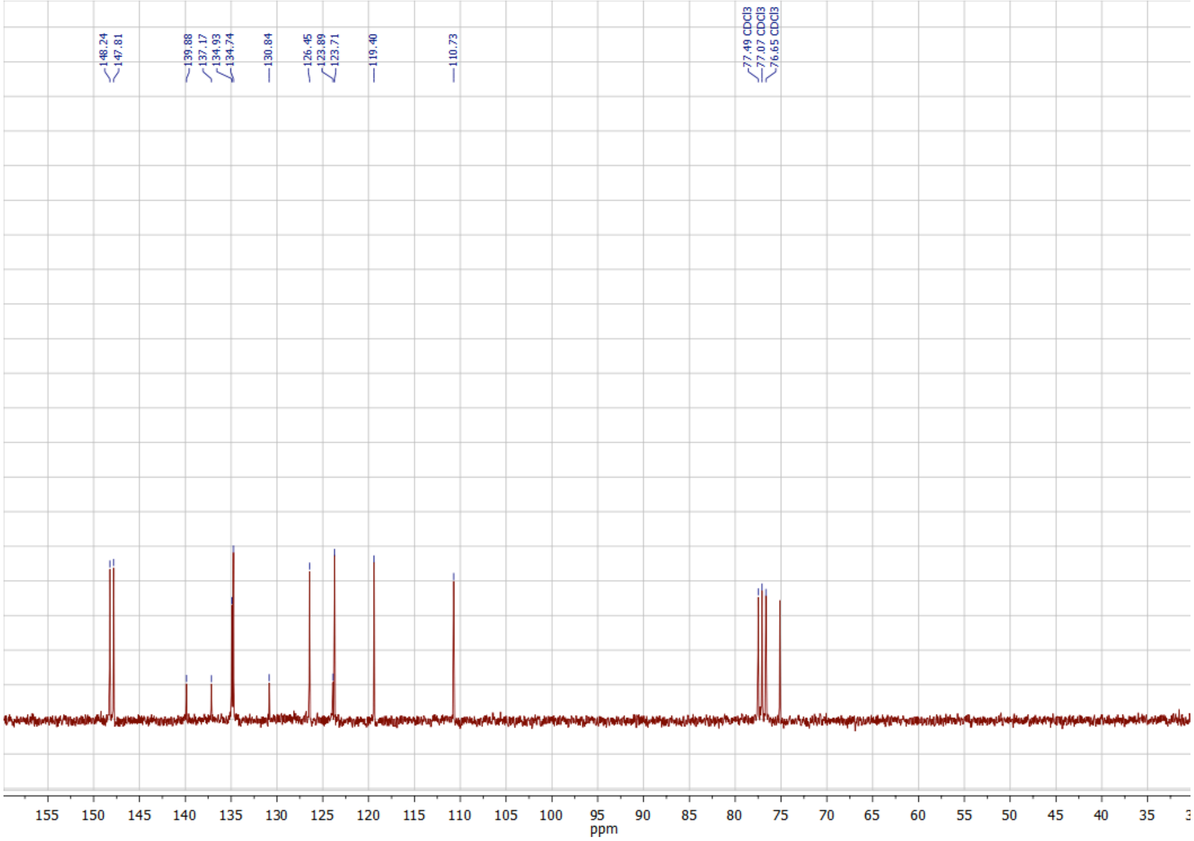

Fig. (S8). ^{13}C NMR spectrum of **10** in $CDCl_3$

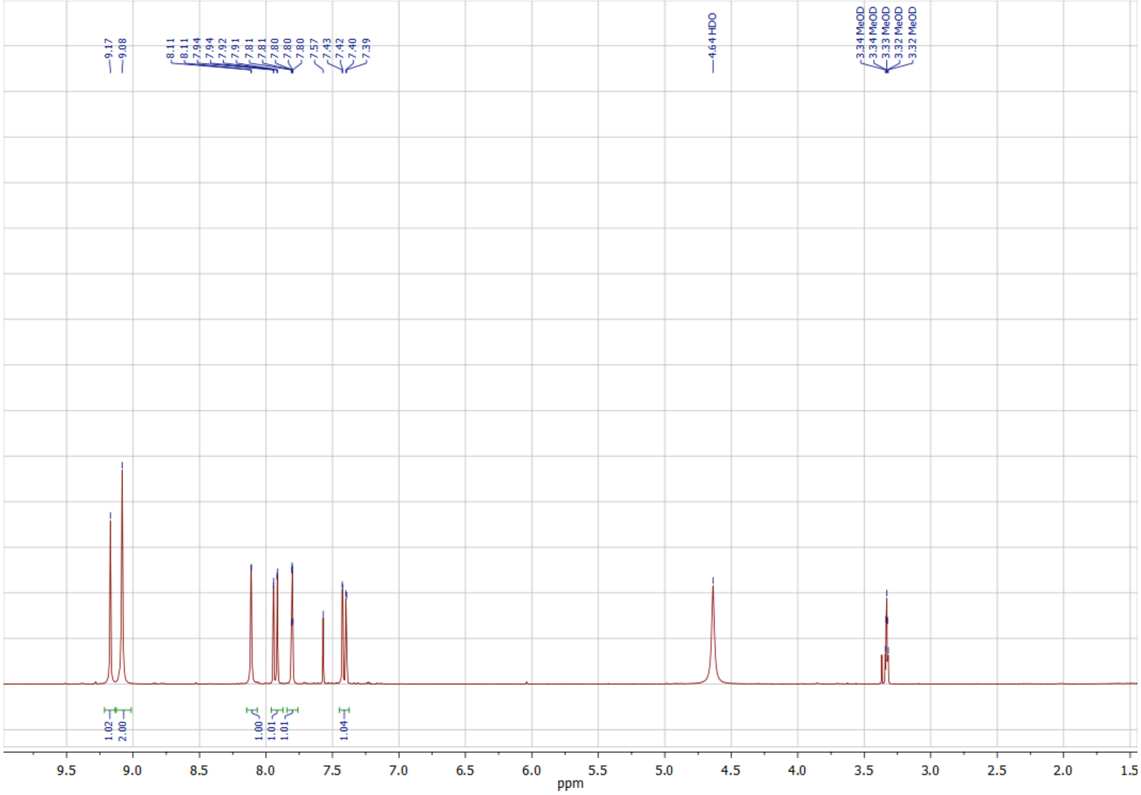

Fig. (S9). ^{1}H NMR spectrum of **11** in CH_3OD

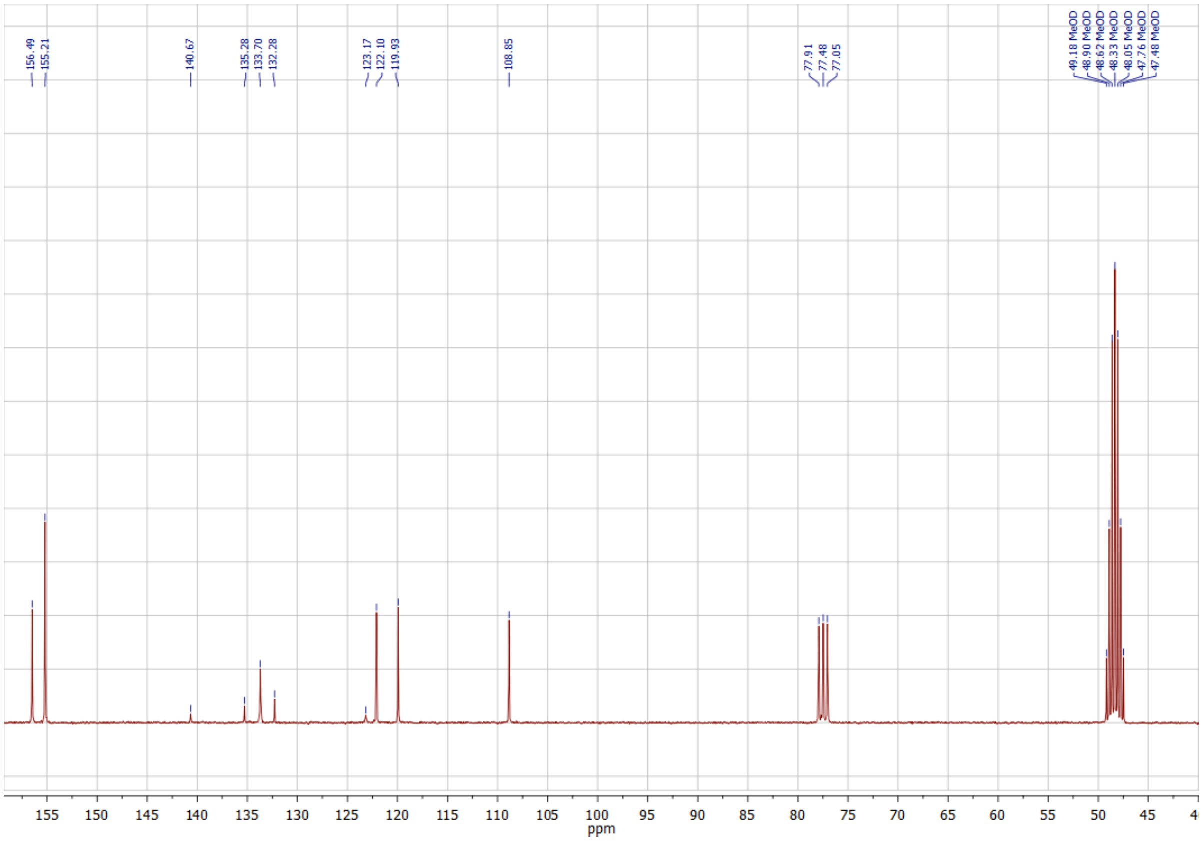

Fig. (S10). ^{13}C NMR spectrum of **11** in CH_3OD.

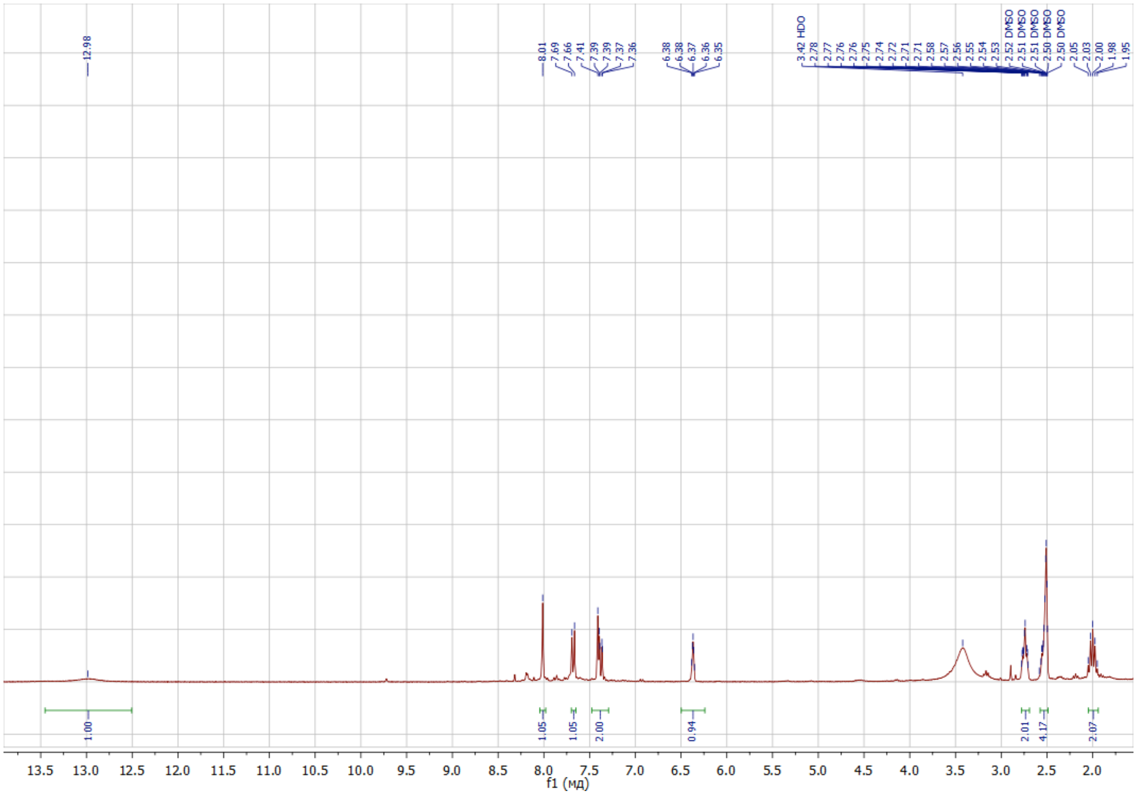

Fig. (S11). ^{1}H NMR spectrum of **12** in $CDCl_3$.

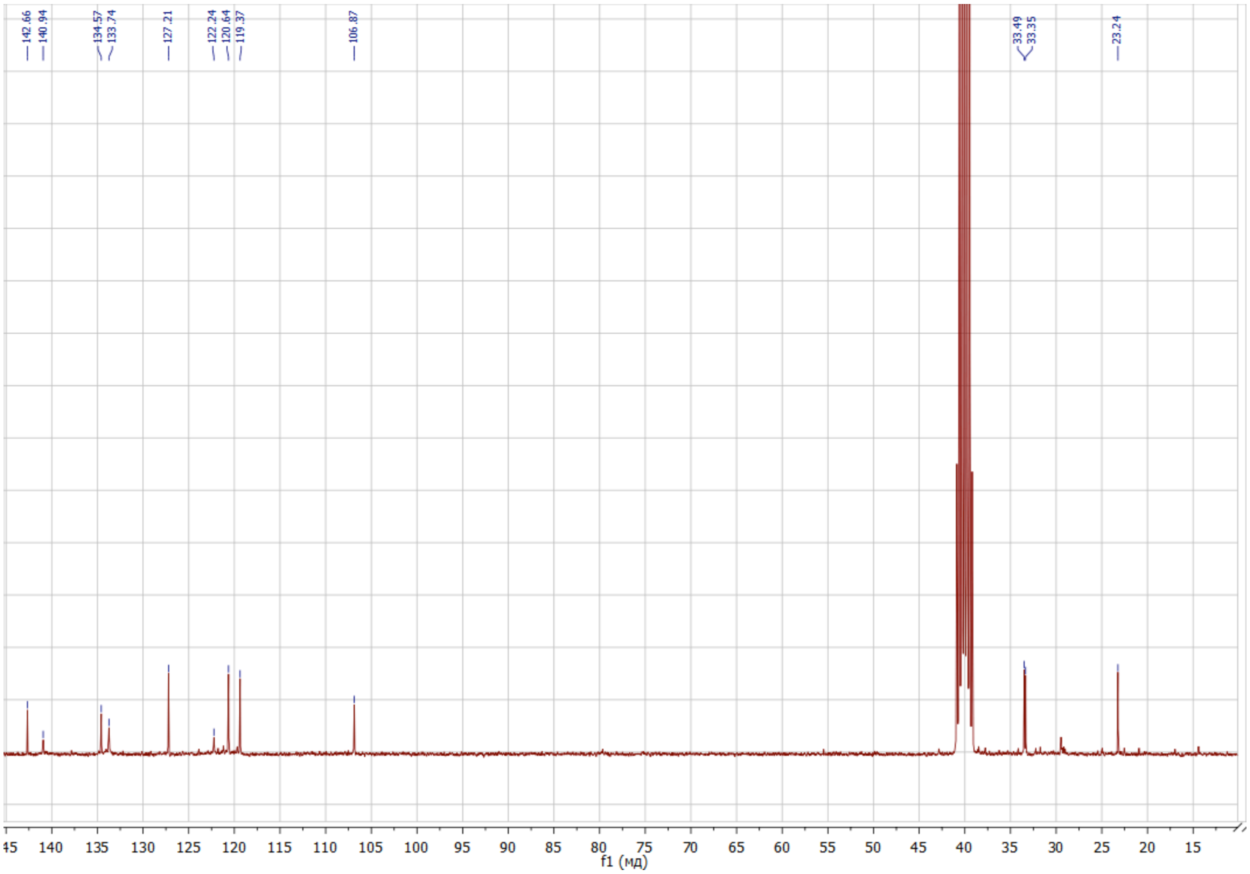

Fig. (S12). ^{13}C NMR spectrum of **12** in $CDCl_3$

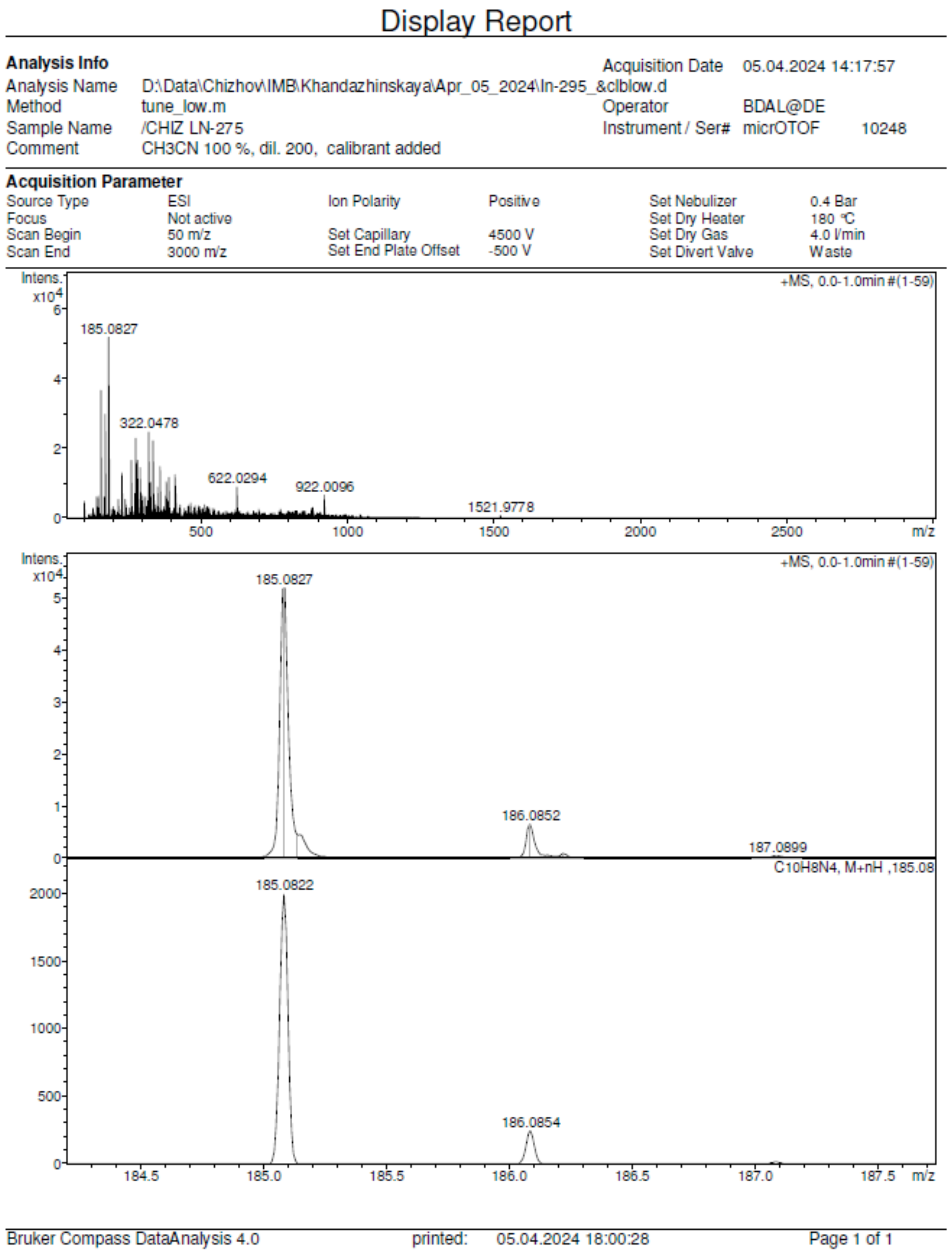

Fig. (S13). HRMS spectrum of **7.**

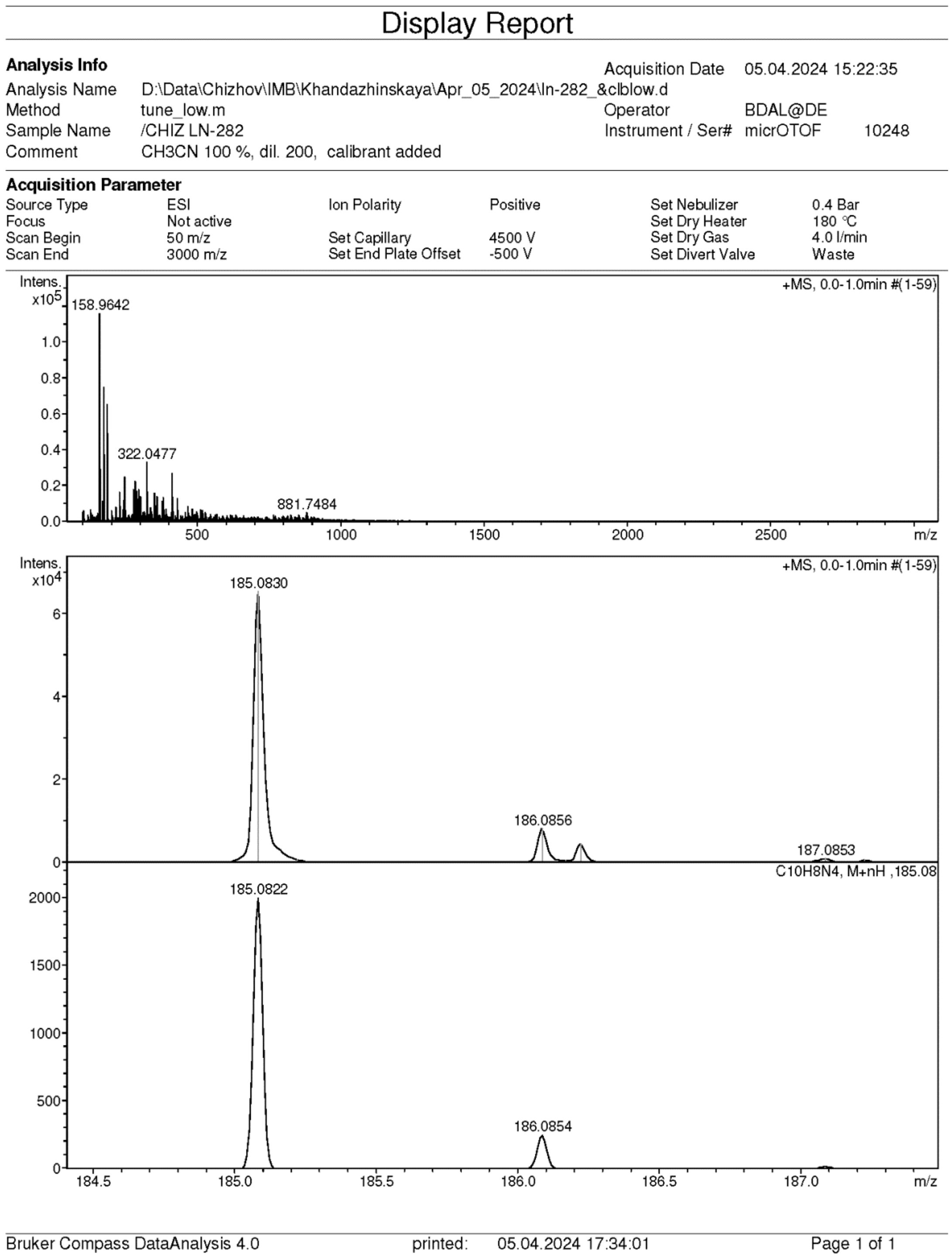

Fig. (S14). HRMS spectrum of **8**

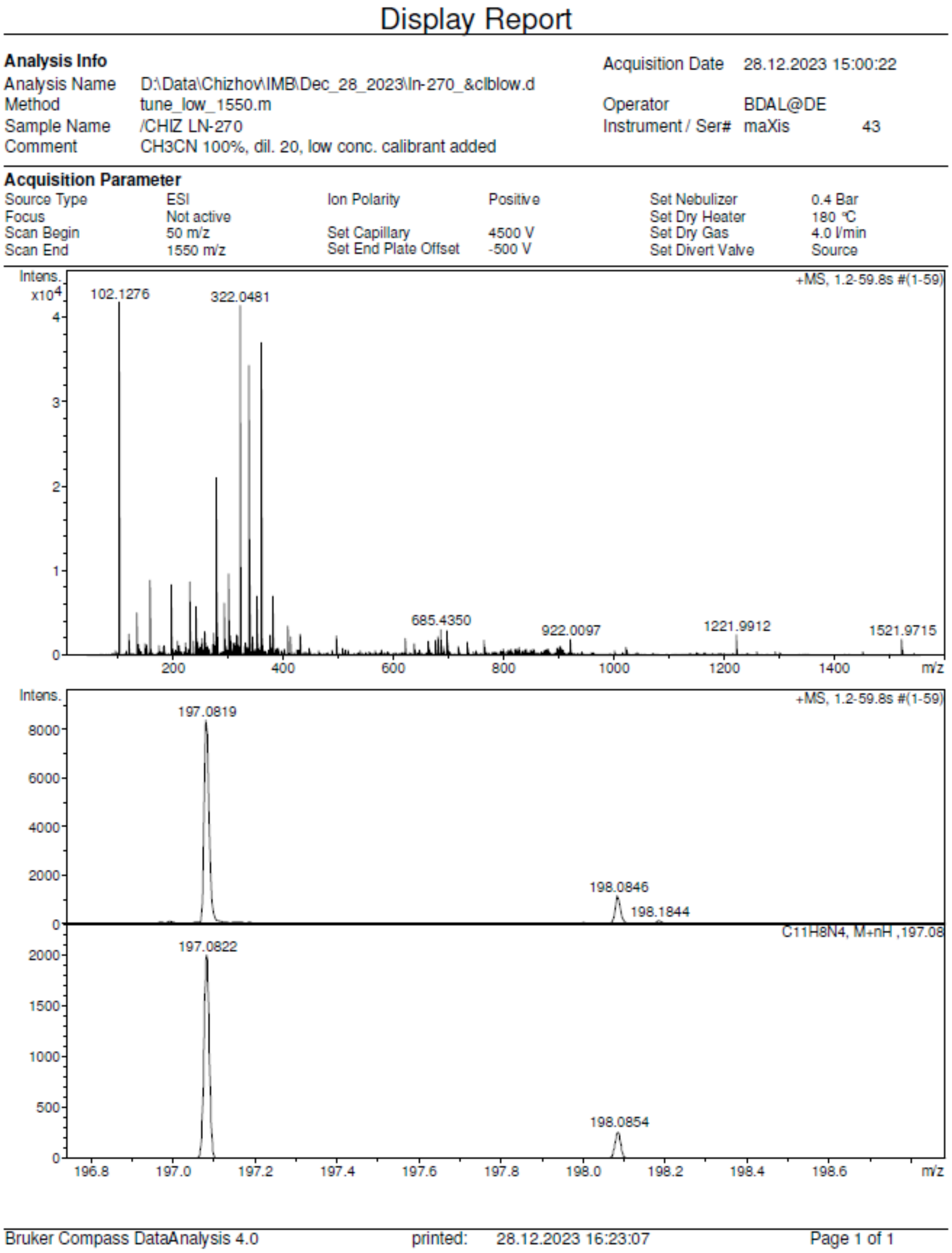

Fig. (S15). HRMS spectrum of **9.**

Display Report

Analysis Info

Analysis Name	D:\Data\Chizhov\IMB\Dec_28_2023\ln-305_&clblow.d	Acquisition Date	28.12.2023 15:05:56
Method	tune_low_1550.m	Operator	BDAL@DE
Sample Name	/CHIZ LN-305	Instrument / Ser#	maXis 43
Comment	CH3CN 100%, dil. 200, low conc. calibrant added		

Acquisition Parameter

Source Type	ESI	Ion Polarity	Positive	Set Nebulizer	0.4 Bar
Focus	Not active			Set Dry Heater	180 °C
Scan Begin	50 m/z	Set Capillary	4500 V	Set Dry Gas	4.0 l/min
Scan End	1550 m/z	Set End Plate Offset	-500 V	Set Divert Valve	Source

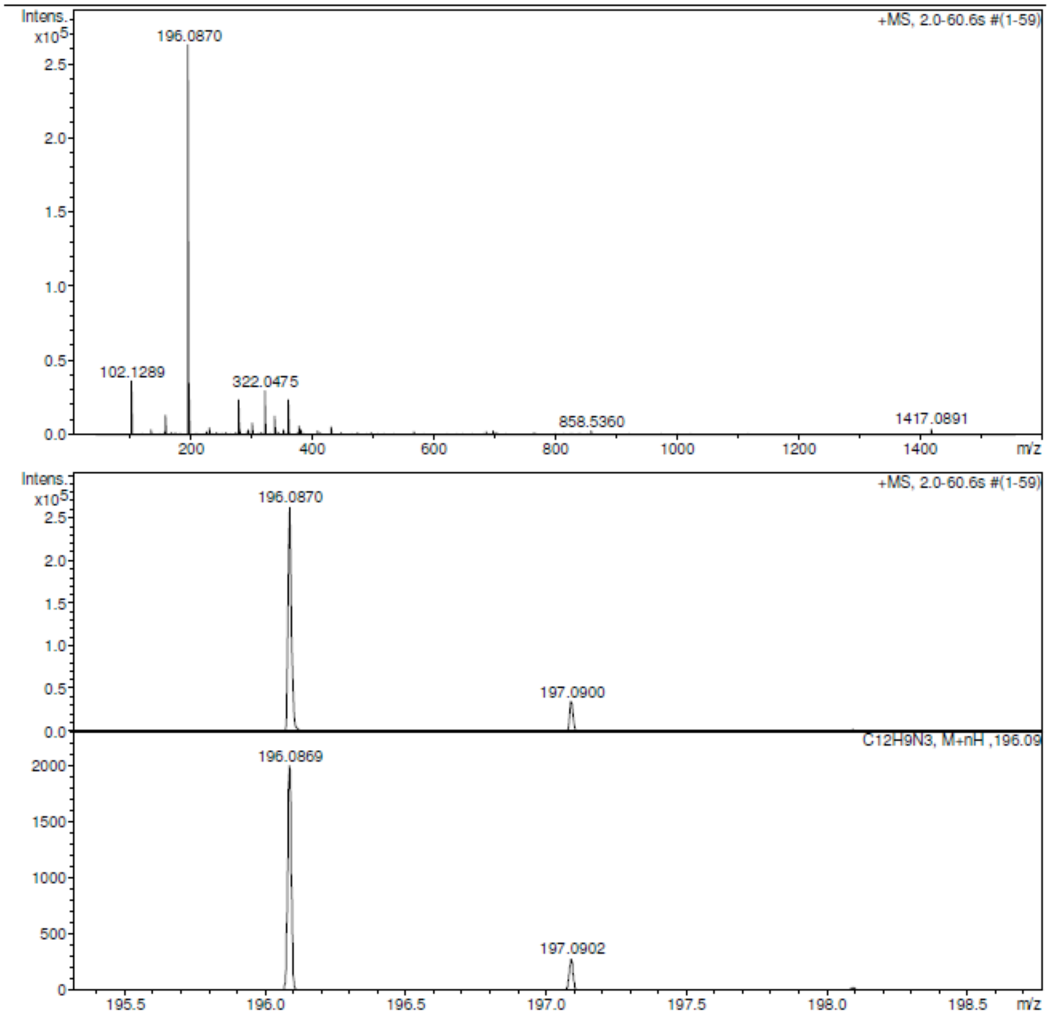

Bruker Compass DataAnalysis 4.0 printed: 28.12.2023 16:25:19 Page 1 of 1

Fig. (S16). HRMS spectrum of **10.**

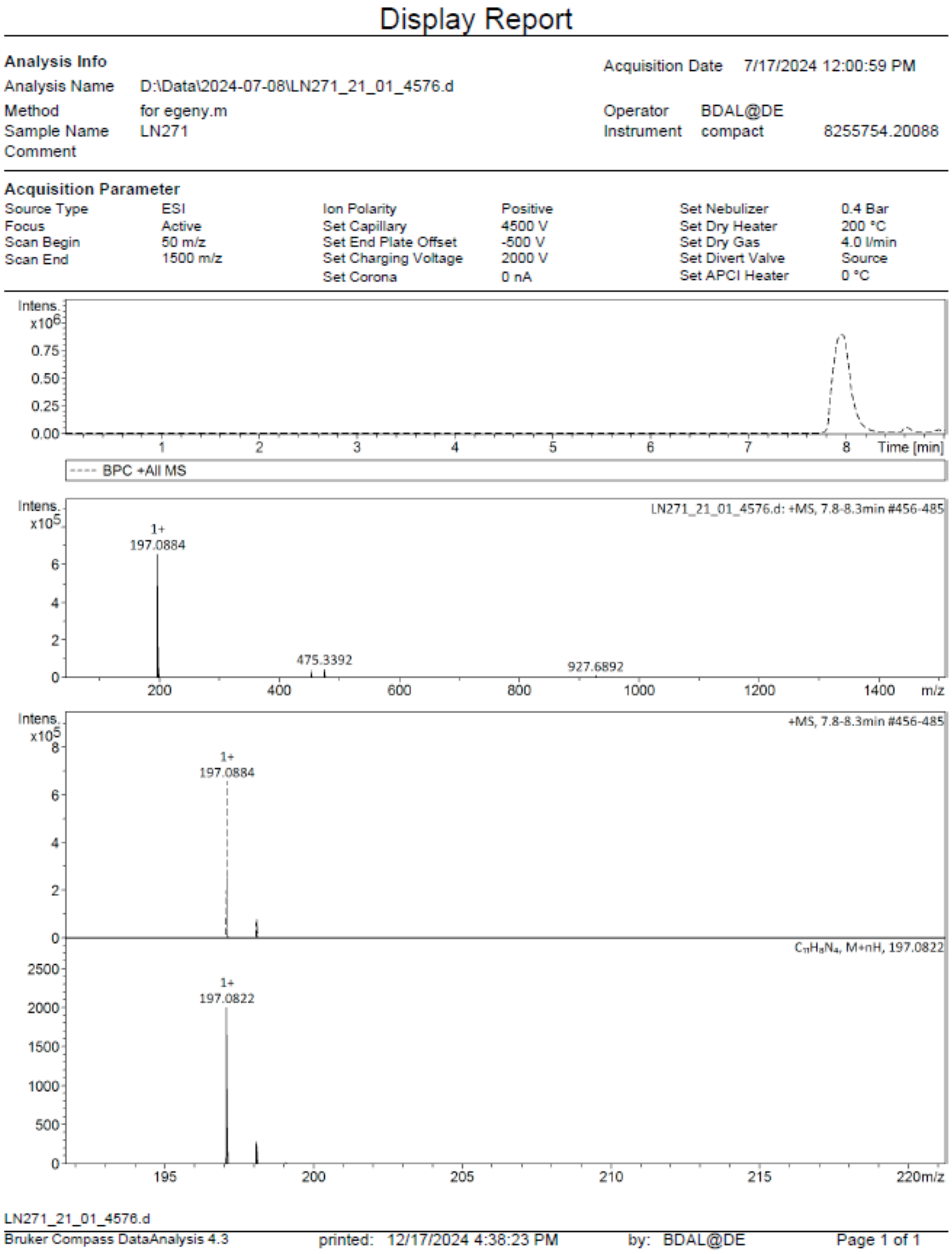

Fig. (S17). HRMS spectrum of **11**.

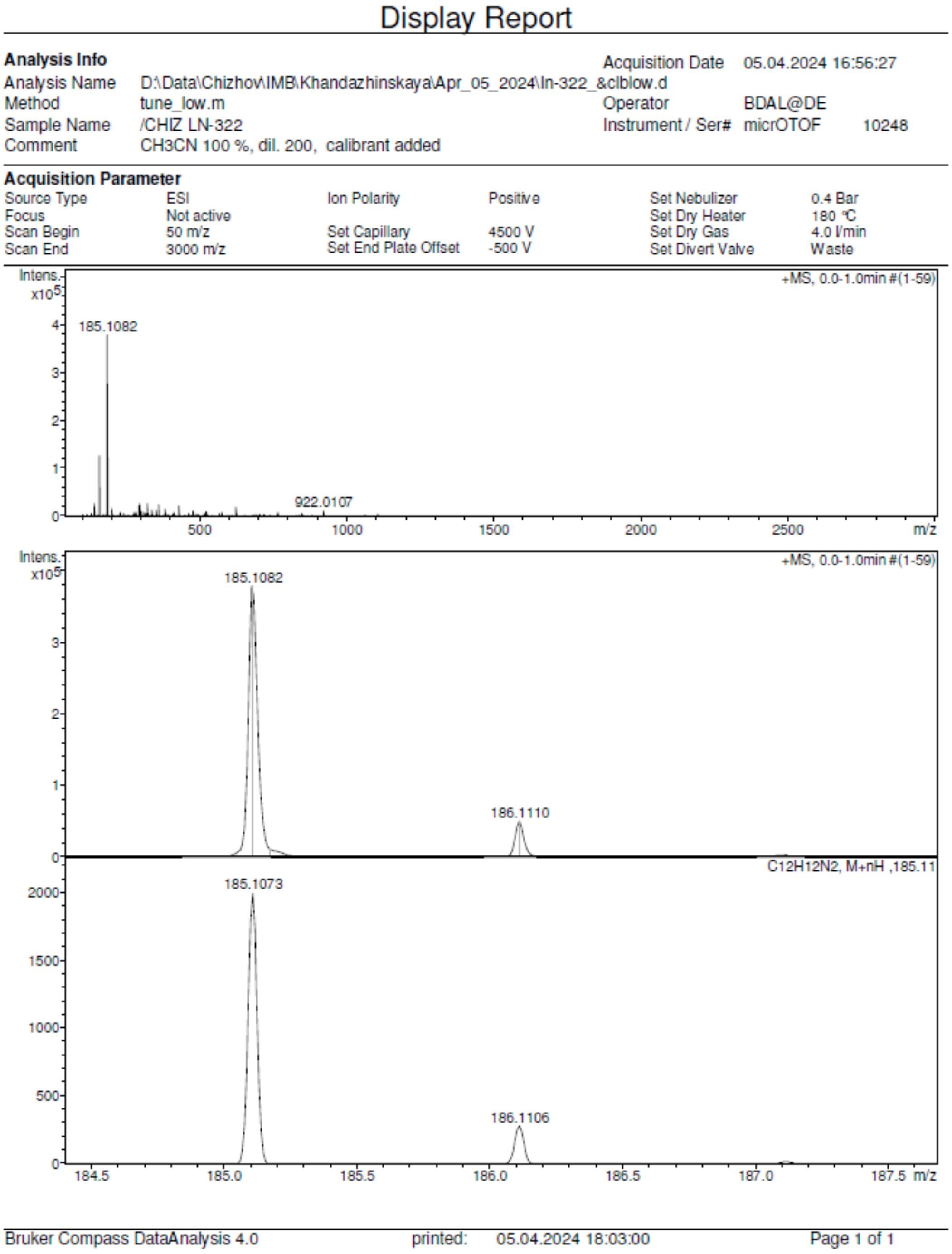

Fig. (S18). HRMS spectrum of **12.**
